# Connexin hemichannel blockade by abEC1.1 disrupts glioblastoma progression, suppresses invasiveness, and reduces hyperexcitability in preclinical models

**DOI:** 10.1186/s12964-025-02370-1

**Published:** 2025-09-02

**Authors:** Viola Donati, Chiara Di Pietro, Luca Persano, Elena Rampazzo, Mariateresa Panarelli, Clara Cambria, Anna Selimi, Lorenzo Manfreda, Ana Gabriela de Oliveira do Rêgo, Gina La Sala, Camilla Sprega, Arianna Calistri, Catalin Dacian Ciubotaru, Guang Yang, Francesco Zonta, Flavia Antonucci, Daniela Marazziti, Fabio Mammano

**Affiliations:** 1 Department of Biomedical Sciences, University of Padua, Padua, 35131 Italy; 2 CNR Institute of Biochemistry and Cell Biology, Monterotondo, Rome, 00015 Italy; 3 Department of Women’s and Children’s Health, University of Padova, Padua, 35128 Italy; 4 Pediatric Research Institute, Padua, 35127 Italy; 5 Department of Medical Biotechnology and Translational Medicine (BIOMETRA), University of Milan, Milan, Italy; 6 Department of Physics and Astronomy “G. Galilei”, University of Padua, Padua, 35131 Italy; 7 Department of Molecular Medicine, University of Padua, Padua, 35121 Italy; 8 CNR Istituto Officina Dei Materiali, Area Science Park Basovizza, S.S. 14, Km 163,5, Trieste, 34149 Italy; 9 Shanghai Institute for Advanced Immunochemical Studies, ShanghaiTech University, Shanghai, 201210 People’s Republic of China; 10 Department of Biosciences and Bioinformatics, School of Science, Xi’an Jiaotong-Liverpool University, Suzhou, 215123 People’s Republic of China; 11 CNR Institute of Neuroscience, Vedano Al Lambro, 20854 Italy

**Keywords:** Glioblastoma, Targeted therapy, Monoclonal antibody, Patient-derived glioma cells, GL261 orthotopic syngeneic mouse model

## Abstract

**Background:**

Connexin (Cx) hemichannels (HCs) contribute to glioblastoma (GBM) progression by facilitating intercellular communication and releasing pro-tumorigenic molecules, including ATP and glutamate.

**Methods:**

The efficacy of abEC1.1, a monoclonal antibody that inhibits Cx26, Cx30, and Cx32 HCs, was assessed in vitro by measuring invasion capability, dye and Ca^2+^ uptake, glutamate and ATP release in patient-derived GBM cultures or organoids. Adeno-associated virus (AAV)-mediated antibody gene delivery, or convection-enhanced delivery (CED) of the purified antibody, was used in vivo to test the effect on tumor growth and animal survival, using a syngeneic GBM mouse model. The ability of the antibody to affect glioma-related hyperexcitability was evaluated by patch-clamp recordings in a 2D co-culture model comprising astrocytes and neurons isolated from mouse hippocampi, seeded with GL261 cells.

**Results:**

abEC1.1 suppressed GBM cell invasion, reducing gliotransmitter release, and impairing tumor progression. In patient-derived GBM cultures, abEC1.1 significantly decreased cell migration and ATP/glutamate release. In vivo, AAV-mediated antibody gene delivery or CED of the purified antibody reduced tumor burden and prolonged survival in the GL261 syngeneic mouse model of GBM. Furthermore, abEC1.1 mitigated glioma-induced excitatory synaptic activity in the 2D co-culture model, suggesting a dual role in tumor control and hyperexcitability suppression.

**Conclusions:**

Our findings establish Cx HC inhibition as a promising therapeutic avenue in GBM and highlight abEC1.1 as a potential candidate for clinical translation.

**Supplementary Information:**

The online version contains supplementary material available at 10.1186/s12964-025-02370-1.

## Abstract (plain language summary)

Glioblastoma is the most aggressive form of brain cancer in adults. It grows quickly, spreads through the brain, and is very hard to treat. A major challenge is that current therapies often fail to stop the tumor’s growth or prevent its return.

This study explores a new approach using a laboratory-made antibody called abEC1.1. This antibody blocks special channels on the surface of tumor cells, called connexin hemichannels, which help the tumor communicate with surrounding cells and release harmful molecules.

The researchers tested abEC1.1 in both human glioblastoma cells and mice with brain tumors. They found that the antibody reduced tumor cell movement, slowed the release of chemicals like ATP and glutamate (which encourage tumor growth and hyperexcitability), and significantly reduced tumor size in mice. The treated mice also lived longer and showed fewer signs of tumor-related brain overactivity, which can lead to seizures.

Importantly, abEC1.1 was effective whether it was delivered as a gene therapy or injected directly into the brain. These findings suggest that targeting connexin hemichannels with abEC1.1 may offer a promising new way to treat glioblastoma and reduce its complications, such as seizures.

**Supplementary Information:**

The online version contains supplementary material available at 10.1186/s12964-025-02370-1.

## Background

Glioblastoma (GBM) is the most aggressive and lethal primary brain tumor in adults, characterized by rapid progression, extensive invasiveness, and profound resistance to standard therapies, including surgery, radiation, and chemotherapy [1]. Despite advancements in therapeutic strategies, the median survival remains approximately 15 months, with fewer than 5% of patients surviving beyond five years post-diagnosis [2]. This stark prognosis underscores the pressing need for novel molecular targets to address GBM’s therapeutic challenges [3].

Connexins (Cxs), a family of 21 transmembrane proteins, have emerged as significant players in cancer biology and as molecular targets with therapeutic potential [4–7]. Cx hemichannels (HCs) facilitate the exchange of molecules between the cytoplasm and the extracellular environment, while gap junction channels formed by the head-to-head connection of two HCs from neighboring cells enable direct cytoplasmic communication [8].

Among Cx subtypes implicated in GBM pathophysiology, Cx26 is a negative prognostic marker in multiple cancers, including glioma [3, 9]. Cx30 contributes to radiation resistance in GBM cells [10], and upregulated Cx32 in activated microglia may influence microglial coordination in the tumor microenvironment [11]. Cx43 is highly expressed in glioma-associated astrocytes, particularly in peri-tumoral regions, where it promotes tumor cell dissemination [12, 13], and recent studies have highlighted the role of Cx43 in GBM invasion through extracellular vesicles (EVs) released by GBM cells [14]. The addition of a selective Cx43 channel inhibitor increased the sensitivity of human O-6-methylguanine-DNA methyltransferase (MGMT)-deficient and temozolomide (TMZ)-resistant GBM cells to TMZ treatment [15]. Glioma stem cells (GSCs), which drive invasion, tumor recurrence, therapeutic resistance [16] and can differentiate into endothelial cells, thereby supporting tumor growth through angiogenesis [17, 18], express elevated levels of Cx46—a condition essential for their maintenance [19, 20].

Other studies have illuminated the pivotal role of calcium (Ca^2+^) and glutamatergic signaling in glioma cell networks [21–26]. Notably, a subset of GBM cells exhibits autonomous, rhythmic Ca^2+^ oscillations, acting as “pacemakers” that drive network activity and promote tumor growth; these periodic Ca^2+^ signals activate frequency-dependent pathways, such as MAPK and NF-κB, which are crucial for tumor progression [27]. Collective invasion by groups of interconnected glioma cells interacting with peritumoral reactive astrocytes increases the likelihood of tumor recurrence [28–30]. Gap junction-mediated intercellular Ca^2+^ waves enhance GBM cell proliferation, migration, survival, and resistance to therapy [31–33]. Elevation of cytosolic Ca^2+^ concentration ([Ca^2+^]_cyt_) in glioma and glial cells promotes release of adenosine triphosphate (ATP) into the extracellular environment through unpaired Cx HCs, with a bell-shaped dependence of HC open probability vs. [Ca^2+^]_cyt_ [34–36]. Extracellular ATP (eATP) acts on purinergic receptors on neighboring cells, further amplifying intercellular Ca^2+^ waves and promoting ATP-induced ATP release [37–39].

eATP is a powerful damage associated molecular pattern (DAMP) that can lead to NLRP3 inflammasome activation, caspase activation, maturation and release of inflammatory cytokines and production of reactive oxygen and nitrogen species (ROS/RNS) [40, 41], all factors that contribute to chronic inflammation, and recruitment and activation of immune cells within the tumor microenvironment (TME) [42]. On the other hand, eATP can also foster immune suppression, angiogenesis and tumor invasion, especially after conversion to adenosine via CD39/CD73 [43–45].

Besides ATP, activated Cx HCs release other gliotransmitters, such as glutamate and D-serine [46–48]. Elevated extracellular glutamate levels in GBM trigger ionotropic and metabotropic receptors that promote neurotoxicity and tumor invasiveness [49–52] as well as glioma-associated seizures, an early pathophysiological hallmark of malignant glioma [53–57]. Glial Cx HCs play a major role in hyperexcitability and epileptogenesis via deregulated Ca^2+^-dependent glutamatergic signaling [40, 58, 59]. Pharmacological inhibition of Cx HCs has been shown to alleviate neuroinflammation and suppress seizure activity in animal models [60–62].

The opening of Cx HC and their capability for autocrine/paracrine signaling [63] also facilitate pathways that drive endothelial cell migration and extracellular matrix remodeling—processes crucial for tumor progression [64, 65]. Genetic knockdown of endothelial connexins impairs angiogenesis [66] and inhibits tumor growth [67]. Boldine, a natural alkaloid, has shown potential to reduce GBM invasiveness [68]. Its effectiveness lies in its ability to target HCs selectively, mitigating their role in promoting a pro-tumorigenic environment without affecting gap junctional communication [69, 70].

The evidence summarized above suggests that targeting Cx HCs represents a promising strategy to disrupt the molecular networks driving GBM pathogenesis [71]. The abEC1.1 monoclonal antibody offers a novel therapeutic avenue by exploiting the druggable nature of these structures [72]. Prior research has demonstrated that abEC1.1 exhibits nanomolar sensitivity and effectively blocks Cx26, Cx30 and Cx32 HC activity [73–78]. In this study, we investigate the therapeutic potential of abEC1.1 by focusing on its ability to inhibit Cx HCs, addressing the critical need for targeted therapies capable of mitigating GBM progression and overcoming its notorious resistance. Unlike previous approaches focusing solely on Cx43, abEC1.1 potentially disrupts broader glioma invasion mechanisms.

## Methods

### Therapeutic antibodies

#### Purified antibodies

Transient transfection of Chinese Hamster Ovary (CHO) cells was used to produce the antibodies employed in this study (Table 1). Antibodies secreted by CHO cells were harvested from the culture supernatant and purified by Protein G affinity chromatography. Purity and molecular weight were assessed by SDS-PAGE (Sodium Dodecyl Sulfate–Polyacrylamide Gel Electrophoresis) under both reducing and non-reducing conditions. SEC-HPLC (Size-Exclusion Chromatography – High-Performance Liquid Chromatography) was performed to assess monomeric content and exclude the presence of aggregates or degradation products. Bacterial endotoxin levels were quantified using the Limulus Amebocyte Lysate (LAL) assay, with all preparations meeting the threshold of < 0.1 EU/mg.

**Table 1:** List of therapeutic antibodies used in this study

#	Name	Type	Use	ALFA-tag
1	abEC1.1(scFv-mFc)	Hybrid, with human scFv and murine Fc	In vitro*:* co-cultures of neurons, astrocytes and GL261 cells	No
2	abEC1.1-mIgG1	Hybrid, with human Fab and murine Fc	In vivo: intratumoral CED	Yes
3	abEC1.1-hIgG1	Fully human IgG1 antibody	In vitro*:* patient-derived cells	No
4	AAV8-abEC1.1(scFv-mFc)	Hybrid, with human scFv and murine Fc	In vivo: antibody gene delivery via ICV injection	No
5	AAV-PhP.Eb-abEC1.1(scFv-mFc)	Hybrid, with human scFv and murine Fc	In vivo*:* antibody gene delivery via RO injection	Yes

1. abEC1.1(scFv-mFc) denotes a single-chain variable fragment (scFv)-fragment crystallizable (Fc) abEC1.1 antibody, consisting of a human scFv (PDB ID: 5WYM) and a murine Fc (mFc) region (Cat. No. B22365001-CHO, Biointron Biological Inc., Shanghai, China). It was utilized in co-cultures of neurons, astrocytes and GL261 cells.
2. abEC1.1-mIgG1 denotes a hybrid full-length antibody, comprising a human antigen-binding (Fab) region, constructed using the same variable heavy and light chain sequences from the scFv fused to a murine immunoglobulin G1 (mIgG1) Fc region carrying a C-terminal ALFA-tag [79] (Cat. No. B22365003-CHO, Biointron Biological Inc.). It was utilized for intratumor administration in vivo via convection enhanced delivery (CED).
3. abEC1.1-hIGg1 denotes a fully human antibody comprising the above-mentioned human Fab region fused to a human immunoglobulin G1 (hIgG1) Fc region [76, 78]. It was generated at the Shanghai Institute for Advanced Immunological Studies of ShanghaiTech University and utilized in experiments with patient-derived glioma cells.

#### Vectored antibodies

4. AAV2 inverted terminal repeats (ITRs) were used to construct a custom expression plasmid (pAAV[Exp]-CAG > [CodOpt-abEC1.1-mFc]:WPRE; Vector ID: VB200421-1436tku; Project Tracking No. T200422-1004wrt, VectorBuilder), encoding abEC1.1(scFv-mFc) under the control of the CAG promoter (a fusion of the cytomegalovirus immediate-early enhancer and the chicken β-actin promoter). Recombinant AAV particles with an AAV8 capsid, denoted as AAV8-abEC1.1(scFv-mFc), were produced using this vector, yielding a final titer of 6.24 × 10^13^ genome copies/mL (Cat. No. AAV8LP(VB200421-1436tku)-C; large-scale AAV8 packaging and ultra-purification service, VectorBuilder). The AAV2 ITR sequence served as the qPCR target for quantification of viral genome copy number following extraction of viral DNA from purified virions. AAV8-abEC1.1(scFv-mFc) were used for in vivo antibody gene delivery via intracereboventricular injection (ICV) (Table 1). AAV8 empty capsids, used as negative controls, were produced and titered by InnovaVector (Naples, Italy).
5. A second construct (pAAV[Exp]-GFAP(short) > :IRES:NLS_TagBFP2:oPRE; Vector ID: VB220714-1064gbp, VectorBuilder) encoding abEC1.1(scFv-mFc) fused to a C-terminal ALFA-tag was packaged using the AAVPHP.eB capsid, yielding a final titer of 1.00 × 10^13^ gc/mL (InnovaVector, Naples, Italy). The resulting viral particles, denoted as AAV-PHP.eB-abEC1.1(scFv-mFc), were used for in vivo antibody gene delivery via retro-orbital (RO) injection (Table 1).

### Patient-derived cell cultures

Primary GBM (hGBM-13 and hGBM-82) cells were isolated from GBM tumors at surgery and cultured as previously described [80]. Briefly, GBM samples were enzymatically and mechanically dissociated into single cell suspensions. Cells were then placed on fibronectin-coated plates and grown as monolayers in DMEM/F12 (Biowest, Nuaillé, France) supplemented with 10% BIT9500 (Stem Cell Technologies, Vancouver, Canada), 20 ng/ml basic Fibroblast Growth Factor (bFGF) and 20 ng/ml Epidermal Growth Factor (EGF; both from Cell Guidance Systems Ltd, Cambridge, UK). GBM cells were maintained in an atmosphere of 2% oxygen, 5% carbon dioxide and balanced nitrogen in a H35 hypoxic cabinet (Don Whitley Scientific Ltd, Shipley, UK) to better resemble the hypoxic conditions of GBM microenvironment [80, 81].

#### RNA isolation and quantitative real-time PCR

RNA was extracted from GBM cells using QIAzol reagent (Qiagen, Hilden, Germany) according to manufacturer’s instructions. Total RNA (1-2 μg) was reverse-transcribed using SuperScript™ First-Strand Synthesis System (Thermo Fisher Scientific, Waltham, MA). Quantitative RT-PCR reactions were run in duplicate using Platinum SYBR Green Q-PCR Super Mix (Thermo Fisher Scientific, Waltham, MA). Fluorescent emission was recorded in real-time (Real Quant Studio 5, Thermo Fisher Scientific, Waltham, MA). The specificity of primers was confirmed for every PCR run by dissociation curve analysis. Primers used are listed in Table 2. Expression values were normalized to the housekeeping gene *GUSB* according to the ΔCt method and expressed as 2^−ΔCt^.

**Table 2:** List of primers used in RT-PCR

Gene	Primer tag	Sequence
*GJB2*	hCx26Fwd	CATGTACGACGGCTTCTCCAT
	hCx26Rev	GCAGGATGCAAATTCCAGACA
*GJB6*	hCx30Fwd	GGACTTCGTCTGCAACACACT
	hCx30Rev	CGAGTGGTTTCGTGCCTGTAG
*GJB1*	hCx32Fwd	CGTGAACCGGCATTCTACTG
	hCx32Rev	TGGTCATAGCAAACGCTGTTG
*GJA1*	hCx43Fwd	GAGCGACCCTTACCATGCGA
	hCx43Rev	TGGTGAGGAGCAGCCATTGA
*GUSB*	hGUSB forward	GAAAATACGTGGTTGGAGAGCTCATT
	hGUSB reverse	CCGAGTGAAGATCCCCTTTTTA

#### Scratch and invasion assays

To evaluate the effects of abEC1.1 on the migratory properties of GBM cells, they were cultured on 12-well plates until they reached 90% confluence. After being gently scratched, GBM cells were washed twice with culture medium to remove cell debris and incubated with abEC1.1-hIgG1 (1 μM or 5 μM) until endpoints (24 h and 48 h). Migrated cells were defined as cells that moved into the scratch. Images were acquired with a Nikon TS100 inverted microscope (Nikon, Melville, NY). Cell migration was evaluated by measuring the distance between the two edges of the scratch in at least 4 random fields/well by using Adobe Photoshop CS6 (Adobe Systems Incorporated, La Jolla, CA).

GBM cells-derived organoids were generated by resuspending 10^5^ cells in 10 µl of Matrigel (Corning, Corning, NY) and plated onto sterile dimpled parafilm mold for the generation of Matrigel droplets as previously described [82]. After Matrigel polymerization (at 37 °C for at least 45 min), organoid droplets were transferred to cell culture dishes in DMEM-F12 medium and allowed to grow for 7 additional days. To assess the invasive capacity of cells, generated 3D GBM structures were plated onto Matrigel-coated 24-well plates and treated with abEC1.1-hIgG1 (1 μM) for 48 h. Images were captured with a Nikon TS100 inverted microscope (Nikon, Melville, NY). Cell invasion was evaluated by measuring the distance covered by invasive GBM cells in the matrix surrounding organoids in at least 8 random fields by using Adobe Photoshop CS6 (Adobe Systems Incorporated, La Jolla, CA).

#### DAPI uptake assay

Cells grown on 12 mm round coverslips were transferred to a spinning disk confocal microscope [83] equipped with a 20 × water immersion objective (Olympus, XLUMPlan Fl, N.A. 0.95).

For control experiments, cells were pre-incubated for 30 min at 37 °C in Ca^2+^-free medium (ZCM) containing (in mM): 138 NaCl, 5 KCl, 0.4 NaH_2_PO_4_, 6 D-Glucose, 10 HEPES (all from Merck), pH 7.3, supplemented with 0.5 mM Ethyleneglycol-bis(β-aminoethyl)-N,N,Nʹ,Nʹ-tetraacetic Acid (EGTA), followed by a 10 min incubation with 5 μM 4′,6-diamidino-2-phenylindole (DAPI) at room temperature in the dark. For pharmacological experiments, cells were similarly pre-incubated for 30 min at 37 °C in ZCM containing 1 μM abEC1.1-hIgG1, followed by the addition of 5 μM DAPI and a 10 min incubation at room temperature in the dark.

DAPI was excited using a 385 nm LED (M385L3, Thorlabs), and emission was collected through a blue band-pass filter (Semrock, FF01-425/26–25) using a cooled sCMOS camera (PCO, EDGE; resolution 2560 × 2160 pixels, 6.5 μm × 6.5 μm, binning 4, 15 s inter-frame interval). To account for DAPI uptake by dead cells, the first image was subtracted from all subsequent images, and DAPI fluorescence traces, Δ*F*(*t*) = *F*(*t*) - *F*(0), were computed as pixel spatial averages from ROIs drawn around the perimeter of at least 150 cell nuclei per field of view (FOV). DAPI uptake rates were estimated by the slope of linear fits to each Δ*F*(*t*) trace.

#### Ca^2+^ uptake assay

For Ca^2+^ uptake, lentiviruses from the pHAGE-RSV-GCaMP6s plasmid (#80,146, Addgene; http://n2t.net/addgene:80146; RRID: Addgene_80146; a gift of Darrel Kotton) were produced and harvested according to standard protocols and used to transduce the cells of interest as previously described [84]. Virally transduced cells expressing the Ca^2+^-selective GCaMP6s indicator [85] and grown on 12 mm round coverslips were pre-incubated for 30 min at 37 °C in ZCM with or without abEC1.1-hIgG1 (1 μM). GCaMP6s was excited by a 470 nm LED (M470L5, Thorlabs), and emission was collected through a 535/30 nm band-pass filter (Cat. No. ET535/30 M, Chroma Technology Corp., Bellows Falls, VT, USA). Sequences of fluorescence images were acquired as described above for 5 min in total (3 s inter-frame interval). Following a 20-s baseline recording, a 15 μL bolus of 200 mM CaCl_2_ was delivered at the edge of the FOV using a 15 μm Ø glass capillary connected to a pneumatic pico-pump (SYS-PV830, World Precision Instruments) to reach a final concentration of 2 mM Ca^2+^ in the bathing solution. Image sequences were analyzed offline using ImageJ, Suite2p [86] and MATLAB (R2019a, The MathWorks). GCaMP6s Δ*F*(*t*) signals were extracted as pixel spatial averages from ROIs drawn around the perimeter of at least 20 cells per FOV. Traces (one per ROI) were corrected for photobleaching by fitting the baseline interval with an exponential decay function and subtracting the extrapolated fit from each trace.

#### Glutamate release assay

The same system and settings used for Ca^2+^ uptake assays were also used to quantify glutamate release, but in this case cells were transduced with a recombinant lentivirus (VectorBuilder, Vector ID: VB210509-1025kym, Catalog # LVM(VB210509-1025kym)-C, Lot # 210628LVZ21, 2.68×10^8^ TU/ml) expressing the intensity-based glutamate-sensing fluorescent reporter iGluSnFR anchored to the plasma membrane on the extracellular side [87]) under the RFPL4b promoter. Virally transduced cells were transferred on the stage of the spinning disk microscope mentioned above and pre-incubated for 30 min at room temperature in (*a*) ZCM, or ZCM supplemented with one of the following: (*b*) 1 µM abEC1.1-hIgG1; (*c*) 200 µM La^3+^; (*d*) 2 mM Ca^2+^. The 15 μm Ø opening of a glass microcapillary filled with the same solution used to pre-incubate the cells (*a, b, c* or *d*) was placed 150 μm above the cells, at the edge of the field of view. Following a 20-s baseline recording, the pico-pump connected to the microcapillary was activated for 1 s to wipe away the glutamate accumulated in the proximity of the cell membrane. The resulting decrease of iGluSnFR fluorescence emission was more or less pronounced depending on the solution used (*a, b, c* or *d*). Fluorescence traces, -Δ*F*(*t*) = *F*(0) - *F*(t), were computed as pixel spatial averages from ROIs drawn around the perimeter of at least 20 cells per field of view. Glutamate release was quantified trace-by-trace as the time integral of -Δ*F*(*t*) over a time interval of 5 min.

#### ATP release assay

ATP release was measured using a luciferin/luciferase assay (ATP Determination Kit, a22066, Invitrogen, Waltham, MA) with a Spark multimodal plate reader (Tecan, Männedorf, Switzerland), according to manufacturer instructions. Cells were seeded in 96-well plates at a density of 1 × 10^4^ cells/well and, after 24 h, incubated at 37 °C for 2 h with or without abEC1.1-hIgG1 (1 μM). ATP released in the media within 5 min from medium replacement was measured by transferring 10 µl of experimental medium into the luciferin/luciferase mix containing white 96-well plates, according to manufacturer’s indications.

### GL261 cell cultures

GL261 cells (ACC 802, Leibniz-Institute, DSMZ-Deutsche Sammlung von Mikroorganismen und Zellkulturen GmbH) were grown in Dulbecco’s Modified Eagle Medium (DMEM, high glucose 4.5 g/L, sodium pyruvate 110 mg/L, cat. n. 11,995–065, Gibco, Thermo Fisher Scientific, MA, USA) complete medium with 10% heat inactivated fetal bovine serum (FBS, cat. n. A56708-01, Gibco), supplemented with 2 mM L-glutamine (cat. n. 25,030–024, Gibco) and 1% penicillin/streptomycin (cat. n. 15,140–122, Gibco). Cells were maintained in an incubator at 37 °C and 5% CO_2_.

### Co-cultures of neurons and astrocytes

Hippocampal neurons and cortical astrocytes were obtained from E18 mice littermates. Dissociated cells were grown at first separately; in particular, hippocampal neurons were plated onto glass coverslips previously coated with poly-L-lysin at the density of 4.5×10^5^ cells per 60 mm Petri dish and grown in neuronal medium containing B27 supplement and glutamate. Astrocytes were plated in 75 cm^2^ flasks in astrocyte culture medium containing 10% FBS. Both neurons and astrocytes were maintained in an incubator at 37 °C and 5% CO_2_.

To obtain neurons-astrocytes co-cultures, cortical astrocytes were shaken and subsequently detached. Astrocytes were counted and plated over days in vitro (DIV) 7–10 neuronal cultures in 60 mm Petri dishes at a density of 5.5×10^5^ cells per dish. Astrocytes were left in co-cultures for about 7 days, necessary to allow the astrocytic leaflet formation, before proceeding with electrophysiological recordings.

### GBM-neurons-astrocytes and GBM-neurons co-cultures

GL261 cells were stored in 90% FBS and 10% DMSO at −80 °C. Before plating, cells were thawed and suspended in 5 mL of GBM culture medium containing 10% FBS; GL261 cells were plated in 25 cm^2^ flasks and maintained in incubator at 37 °C and 5% CO_2_.

To obtain GBM-neurons-astrocytes and GBM-neurons co-cultures, GL261 cells were detached and plated on neuronal-astrocytes co-cultures or on pure neurons in 60 mm Petri dishes at the density of 7.5 × 10^4^ per dish. GL261 cells were left in co-cultures for 3 h before proceeding with electrophysiological recordings. A longer co-culture period was not feasible as neurons exhibited evident signs of degeneration and cell death already 6 h after GL261 seeding (Fig. S8).

#### Electrophysiological recordings in GBM-neurons-astrocytes co-cultures

Whole-cell patch-clamp recordings were obtained in the voltage-clamp modality [88] using the Axopatch 200B amplifier and the pClamp-10 software (Axon Instruments). Recordings were performed in Krebs’-Ringer’s-HEPES (KRH) extracellular solution (NaCl 125 mM, KCl 5 mM, MgSO4 1.2 mM, KH2PO4 1.2 mM, CaCl_2_ 2 mM, glucose 6 mM, HEPES–NaOH pH 7.4 25 mM). Recording pipettes were fabricated from glass capillaries (World Precision Instrument) using a two-stage puller (Narishige) and were filled with a potassium-gluconate intracellular solution (KGluc 130 mM, KCl 10 mM, EGTA 1 mM, HEPES 10 mM, MgCl_2_ 2 mM, MgATP 4 mM, GTP 0.3 mM). Pipettes had a tip resistance of 3–5 MOhm. To identify mEPSCs, neurons were held at −70 mV and tetrodotoxin (TTX, 1 μM) was added to the extracellular solution. Multi-unit (MU) events were recorded in the cell-attached configuration at a holding potential of −50 mV in KRH without TTX. Currents were sampled at 10 kHz and filtered at 2 kHz. Recorded traces were analyzed offline using the Clapfit/pClamp-10 software, after choosing an appropriate threshold. Recordings of basal neuronal activity were performed in DIV14 hippocampal neurons, either in pure neuronal cultures or co-cultured with astrocytes (astrocyte: neuron ratio = 1.5). To investigate the effect of the antibody, after collecting control traces, cultures were incubated for 45 min with 1 µM abEC1.1(scFv-mFc) and a new set of traces was acquired from the same culture. Likewise, to investigate the effects mediated by GL261 cells, after collecting control traces, GL261 cells were plated on neuronal-astrocytic co-cultures or on pure neuron cultures at a density of 7.5×10 cells per dish and electrophysiological recordings were performed 3 h later. Finally, abEC1.1(scFv-mFc) was administered in the triple co-culture (GBM-neurons–astrocytes) or in the GBM-neurons co-culture and recordings performed after 45 min of incubation with the antibody (1 µM).

### Animal strains, housing and care

C57BL/6 J mice were provided and bred by Consiglio Nazionale delle Ricerche-European Mouse Mutant Archive (CNR-EMMA)-Infrafrontier specific pathogen-free (SPF) unit (Monterotondo Scalo, Rome, Italy).

After weaning, mice were housed by litter of the same sex, 3 to 5 per cage in ventilated cages at a temperature of 20 ± 2 °C, relative humidity of 55 ± 15% with 12–15 air changes per hour and a 12/12-h light/dark cycle. Certified dust-free wood bedding (Scobis one, Mucedola, Settimo Milanese, Milan, Italy) was provided in the cages. Mice were fed a standardized mouse diet (4RFN and Emma 23, Mucedola) and were provided chlorinated, filtered water ad libitum.

Treated mice, both males and females, and relative controls were individually housed and observed daily.

#### Anesthesia

Adult C57BL/6 J mice (P75) were anesthetized with isoflurane (induction 5% isoflurane, maintenance 2% isoflurane; 0.6 L/min) and maintained at 37 °C on a controlled heating pad. For pain control, buprenorphine was administrated subcutaneously (0.1 mg/Kg body weight).

P0.5 pups were anaesthetized similarly while being maintained in an ad hoc-designed apparatus [89].

#### In vivo syngeneic intracranial tumor induction

GL261 cells were grown to 70% confluence, harvested using 0.05% Trypsin at 37 °C for 3 min and resuspended in DMEM complete culture medium. Shortly before implantation, cells were sedimented by centrifugation at 1000 rpm for 3 min and resuspended in Hanks′ Balanced Salt solution (HBSS, cat. n. 14,025–050, Gibco), counted with an automated cell counter (Countess, cat. n. C10227, Invitrogen, Thermo Fisher Scientific, MA, USA) and resuspended in HBSS.

Adult (P75) C57BL/6 J mice were anaesthetized and the head was secured with ear bars to the head holder of a stereotaxic apparatus (cat. n. 68,513, RWD, TX, USA). Two μl of GL261 cell suspension containing either 2×10^4^ or 3×10^4^ cells/µL were delivered at a speed of 1 nl/s through a 0.5 mm craniotomy performed at the following coordinates from bregma: intracortical injections, AP = −1 mm, ML = + 1.5 mm, DV = −1.1 mm; intrastriatal injections, AP = + 0.86 mm, ML = + 1.8 mm, DV = −3 mm. For these operations, we used beveled glass capillaries with 100 μm diameter aperture mounted on a 100 µL NanoFil (World Precision Instruments, WPI, FL, USA) syringe prefilled with mineral oil (cat. n. M5904, Merck, MA, USA) connected to a microinjection pump (PhD Ultra 70–3601 Nanomite Syringe Pump, Harvard Apparatus, MA, USA). After cell delivery, the capillary was left in place for 15 min and withdrawn slowly to avoid fluid spillover. The hole was sealed with bee wax and the wound was sutured.

### Antibody gene delivery

#### ICV injection of AAV8-abEC1.1(scFv-mFc) in postnatal day (P)0.5 pups

ICV injections were performed on anaesthetized P0.5 pups using a 33-gauge beveled needle held in a 100 µL NanoFil (WPI) syringe connected to a microinjection syringe pump (UMP3 UltraMicroPump, WPI) mounted on a stereotaxic apparatus. Two µL of AAV8-abEC1.1(scFv-mFc) (6×10^10^ gc/µL) dispersed in PBS were delivered into the lateral ventricle of the right hemisphere (coordinates from lambda: ML = + 0.8 mm, AP = + 1.5 mm and DV = − 3 mm). Pups were then placed over a warming pad at 37° C until awaking and returned to their mother’s cage.

#### RO injection of AAVPHP.eB-abEC1.1(scFv-mFc) in P75 mice

The day after intracortical implantation of GL261 cells, mice were anesthetized and maintained at 37 °C on a controlled heating pad. RO injection of 100 μl of AAV-PHP.eB-abEC1.1(scFv-mFc) (3×10^11^ gc) diluted in saline solution was performed according to standard procedures [90].

### Antibody CED

Mice previously implanted with GL261 cells in the striatum were anesthetized and 10 µL of artificial cerebrospinal fluid (ACSF: NaCl 120 mM, KCl 2.5 mM, MgCl_2_ 1 mM, NaHPO_4_ 1.2 mM, CaCl_2_ 2 mM, NaHCO_3_ 26 mM, D-Glucose 11 mM; pH 7.2–7.4) or 10 µL of ACSF supplemented with 5 mg/mL of abEC1.1-mIgG1 were injected through the same craniotomy used for the intrastriatal implantation GL261 cells. For these operations, we used beveled glass capillaries with 100 μm diameter aperture mounted on a 100 µL NanoFil syringe (WPI) prefilled with mineral oil connected to a microinjection pump (PhD Ultra 70–3601 Nanomite Syringe Pump, Harvard Apparatus) at the following rates: 3 µL at 0.2 µL/min followed by 5 µL at 0.5 µL/min and 2 µL at 0.8 µL/min. To avoid fluid spillover, the capillary was then left in place for 5 min and finally withdrawn slowly. The hole was sealed with bee wax and the wound was sutured.

### Immunofluorescence staining of mouse brain sections

Mouse brains were dissected, and post-fixed overnight in 4% paraformaldehyde (PFA, cat. n. P6148, Merck) in 1 × PBS (cat. n. 161–0780, Bio-Rad, CA, USA) at 4 °C. Upon embedding in 2% agarose (cat. n. A0575, Merck), the entire tissues were serially sectioned (50 μm coronal sections) using a Vibratome (VT1000 S, Leica Biosystems, IL, USA).

For immunostaining, sections were permeabilized with 0.5% Triton X-100 (cat. n. 108,603, Merck) in 1×PBS for 1 h at room temperature, incubated for 1 h in blocking buffer containing 5% normal donkey serum (cat. n. S30, Merck), 3% bovine serum albumin (BSA; cat. n. A4503, Merck) and 0.05% Tween-20 (cat. n. 1,706,531, Bio-Rad) in 1×PBS. Samples were then incubated overnight at 4 °C, with primary antibodies (listed in Table 3) diluted in blocking buffer, followed by washing and incubation with fluorophores-conjugated secondary antibodies (listed in Table 4). Nuclei were stained 4,6-diamino-2-phenylindole (DAPI; cat. n. D1306, Molecular Probes) and microscope slides were mounted with Prolong (cat. n. P36934, ThermoFisher Scientific).

**Table 3:** List of primary antibodies used in immunohistochemistry

Name	Company	Species	Cat. Number	Dilution
ALFA-tag	NanoTag	Rabbit; pAB	N1583	1:200
Cx26	TFS	Mouse; mAB	13–8100	1:100
Cx30	TFS	Rabbit; pAB	71–2200	1:50
Cx43	TFS	Rabbit; pAB	71–0700	1:50
Cx46	TFS	Rabbit; pAB	38–8300	1:50
Gfap	Pharmingen	Mouse; mAB	556,327	1:50
Gfap	Synaptic System	Rabbit; pAB	173 002	1:1000
Ki-67	TFS	Rabbit; pAB	PA5-19,462	1:100
Iba1	Wako Chemicals	Rabbit; pAB	\	1:500
PH3	TFS	Rabbit; pAB	PA5-17,869	1:100
β_III_ Tubulin	Promega	Mouse; mAB	G712A	1:250

*Abbreviations:* *TFS* ThermoFisher Scientific, *mAB* monoclonal antibody, *pAB* polyclonal antibody

**Table 4:** List of secondary antibodies used in immunohistochemistry

Name	Company	Cat. Number	Dilution
Donkey anti-Rabbit, Alexa Fluor 555	TFS	A-31572	1:800
Donkey anti-Mouse, Alexa Fluor 555	TFS	A-31570	1:800
Goat anti-mouse Alexa Fluor 488	TFS	A-11029	1:800
Donkey anti-Rabbit, Alexa Fluor 488	TFS	A-21206	1:800

For detection of abEC1.1 downstream of AAV8-abEC1.1(scFv-mFc) ICV injection using the anti-ALFA-tag antibody, animals were subjected to intracardiac perfusion of 30 mL 1 × PBS, 2000 UI/mL heparin (cat. n. H3149, Merck), followed by 25–30 mL ice-cold 4% PFA. Brains were immediately removed and postfixed in a 4% PFA fixative solution overnight at 4 °C. Vibratome sections, prepared as described above, were permeabilized with 0.3% Triton in 1×PBS for 1 h at room temperature and incubated overnight with Donkey anti-mouse Alexa488 diluted 1:300 in 3% donkey serum and 0.03% Tween-20 in 1×PBS. DAPI staining of nuclei and microscope slide preparation was performed as indicated above.

### Immunofluorescence in vitro staining on neuronal co-cultures

Neurons–astrocytes co-cultures and GBM–neurons–astrocytes co-cultures were fixed in 4% paraformaldehyde and 4% sucrose for 20 min, then washed with 1 × PBS. This was followed by three washes with a low-salt solution (150 mM NaCl and 10 mM phosphate buffer, pH 7.4) and three washes with a high-salt solution (500 mM NaCl and 20 mM phosphate buffer, pH 7.4). Slices were incubated for 30 min with 1 × GSDB (Goat Serum Dilution Buffer: 16.7 × Goat Serum, 20 mMphosphate buffer pH 7.4, 450 mM NaCl, and 0.3 × Triton X-100) for permeabilization, and subsequently incubated for 3 h with primary antibodies (listed in Table 2) diluted in 1 × GSDB at optimized concentrations. After primary incubation, slices were washed with the high-salt solution and then incubated with fluorophores-conjugated secondary antibodies (listed in Table 3) for 1 h. Nuclei were stained with DAPI and microscope slides were mounted with Mowiol (cat. n. 81,381, Merck). Representative images were acquired using the Nikon Eclipse Ni fluorescent microscope with a 20 × objective and the Stellaris 8 confocal microscope with a 10 × objective.

### Quantitative analysis of mouse brain sections

#### Immunofluorescence image acquisition and analysis

Fluorescence micrographs were acquired with a TCS SP5 confocal microscope (Leica Microsystems) equipped with a 40 × oil immersion objective (HCX PL Apo, UV optimized, N.A. 1.25, oil, Leica Microsystems) using Leica Application Suite Advanced Fluorescence 2.7.3.9723 (LAS AF) software. Alexa Fluor 488 and GCaMP6s fluorescence was excited by a 488-nm Argon laser and collected between 500 and 540 nm. Alexa Fluor 555 fluorescence was excited by a 543-nm HeNe laser and collected between 568 and 680 nm. DAPI fluorescence was excited by a 405-nm Diode laser and collected between 441 and 487 nm.

Lower magnification images for quantification of tumor volume and abEC1.1-mIgG1 expression after CED were acquired with a motorized Leica LMD7000 Microdissection System (Leica Microsystems) using the manufacturer’s imaging software (Leica application suite X, 3.6.0.20104).

#### Tumor volume estimation

Sections containing brain tumors were identified by DAPI staining and tumor volumes were estimated using the Fiji/ImageJ analysis software [91] by summing all tumor-positive areas multiplied by the slice thickness (50 μm) along the entire sectioning axis.

#### Quantification of proliferation marker indices

Mosaic images of sections containing brain tumors and labelled with anti-Ki-67 or ant-PH3-antibodies were acquired with the TCS SP5 confocal microscope. All DAPI-stained nuclei were counted using QuPath 4.3 software [92] with StarDist extension [93]. The nuclei that were positive to the proliferation marker were also counted and the Ki-67 index or PH3 index was calculated as the ratio between the Ki-67 or PH3 positive cells and the total number of nuclei.

#### Quantification of abEC1.1-mIgG immunoreactivity following CED

Images from brain slices of mice, treated with CED of abEC1.1-mIgG labelled with an anti-ALFA-tag antibody, or saline solution, were acquired with the LMD7000 Microdissection System using the manufacturer’s image acquisition software. To quantify ALFA-tag immunoreactivity using Fiji/ImageJ analysis software, background levels were evaluated as the mean intensity inside a region of interest (ROI) drawn in an unlabeled brain area and subtracted. Then, for each slice a circular ROI with a radius of 100 pixels was selected within the tumor mass. Another ROI of the same size was selected in the adjacent peritumoral area and the mean fluorescence of the secondary antibody recognizing the anti-ALFA-tag antibody within each ROI was computed.

#### Quantification of Gfap- and Iba1-positive relative areas

Quantification was performed using Fiji/ImageJ Images on sections containing brain tumors, labelled with anti-Gfap or anti-Iba1-antibodies and acquired with the TCS SP5 confocal microscope. Gfap- and Iba1-positive pixels were identified by thresholding and their number was divided by that of the corresponding tumoral or peritumoral regions.

### Statistics

Statistical analyses were performed using MATLAB (R2019a, The MathWorks) and graphpad Prism 8.01. The normality of data distributions was assessed with the Shapiro–Wilk test. For normally distributed data, comparisons of means were conducted using ANOVA, followed by Bonferroni post hoc tests or Dunnett’s multiple comparisons test for multiple group comparisons or two-tailed t-tests for comparisons between two groups. For non-normally distributed data, the Kruskal–Wallis (KW) test was used, with Dunn-Sidak post hoc tests for multiple group comparisons. Results are presented as dot-box plots. Statistical significance was defined as *p*-values (p) less than 0.05. Sample sizes (n) for each experimental group are indicated in the figure legends and were determined based on feasibility considerations as this is an exploratory study aimed at generating new hypotheses and identifying response trends not described in the literature.

In animal studies, mice were randomly assigned to experimental or control groups using a computer-generated randomization sequence (simple randomization without stratification). The experimenters responsible for animal implantation and treatment administration were not involved in group allocation. In vitro experiments involving patient-derived GBM cultures were conducted in parallel for treated and untreated groups using cells from the same culture batch. Cultures were randomly allocated to treatment conditions based on their location in multi-well plates to minimize positional bias.

For all animal experiments, outcome assessment was performed by investigators blinded to group allocation. Tumor volume quantification, immunofluorescence analysis, and histological evaluations were conducted using coded samples. In vitro outcome analyses (e.g., cell migration, DAPI and Ca^2+^ uptake, ATP/glutamate release) were carried out by investigators who were unaware of the experimental conditions until the final statistical analysis.

All animals and samples that met the predefined inclusion criteria were included in the analysis. Animals were excluded only in cases of procedural failure (e.g., unsuccessful intracranial injection or antibody delivery), unexpected death unrelated to tumor burden, or poor tissue preservation that precluded histological analysis. No animals or data points were excluded post hoc based on experimental outcome. In vitro data points were excluded only if they showed signs of culture contamination or poor viability prior to treatment. All endpoints and outcome measures collected during the study are reported in the results. No experimental results that contradicted the main findings were omitted from the manuscript.

## Results

### abEC1.1 is effective against patient-derived GBM cell cultures

Interrogation of publicly available datasets revealed various levels of expression of Cx genes in human GBM tumor biopsies, with Cx43 taking a leading role (Fig. S1a). This analysis also confirmed the reduced survival rates of patients with elevated Cx26 in GBM (Fig. S1b) whereas survival was independent of Cx43 expression level (Fig. S1c).

Using qPCR, we quantified Cx expression levels in patient-derived primary cell cultures. In two of them, hereafter referred to as hGBM-13 and hGBM-82, we detected higher-than-average levels of Cx26 and Cx32 (Fig. S2), which are proven targets of the abEC1.1 antibody [73–78].

**Fig. 1:**
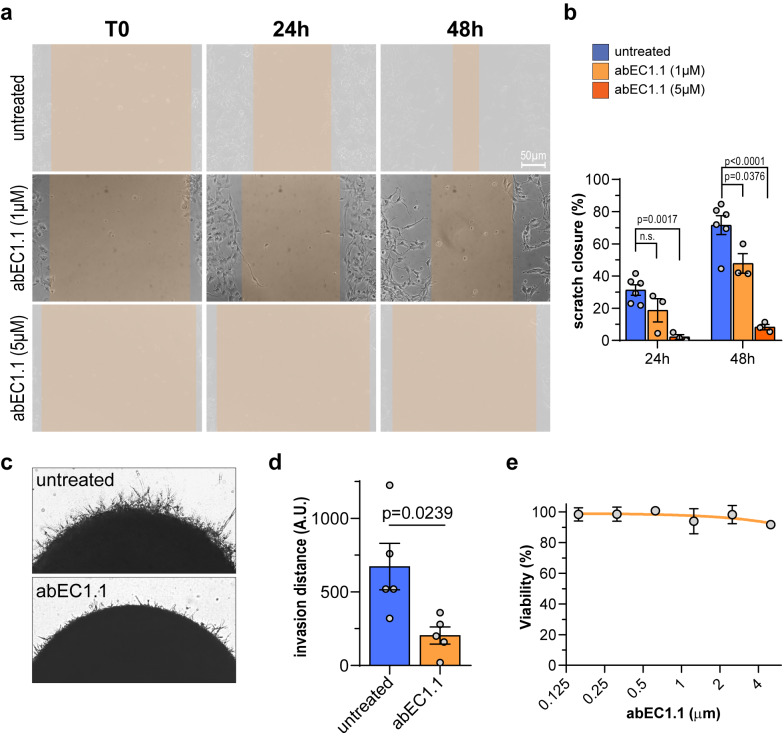
Scratch, invasion and antibody toxicity assays in patient-derived hGBM-13 cell cultures. **a** Representative images displaying the effect exerted by increasing doses of abEC1.1 on the ability of hGBM-13 primary cells to close the scratch over time. Scale bar: 50 µm. **b** Bar-dot plots of percent scratch closure data from (**a**); pooled data from *n* = 3 independent experiments. *P*-values (p) determined by One-way ANOVA with Dunnett’ multiple comparisons test. **c** Representative images displaying the ability of control or abEC1.1 (1 µM)-treated hGBM-13 organoids to invade Matrigel in 48 h. **d** Bar-dot plots of cell invasion quantification, measured by the mean distance between invasive cells and organoid edges; pooled data from *n* = 4 independent experiments. **e** Dose response curves showing the effect of the administration of scalar doses of abEC1.1 on hGBM-13 cell viability, through a resazurin-based assay

We then treated these primary cultures with abEC1.1 and assessed its effect using scratch and invasion assays (Fig. 1 and Fig. S3). Scratch assays provide insights into the mechanisms and factors influencing cell migration, including the role of cell signaling, extracellular matrix interactions, and therapeutic interventions [94]. Invasion assays help identify molecules and pathways that drive invasive behavior, aiding in the development of targeted therapies [95]. Compared to untreated controls, treatment of hGBM-13 cell cultures with abEC1.1 for 24 or 48 h resulted in a significant dose-dependent reduction in cell migration (Fig. 1a, b) and invasion (Fig. 1c, d) with no appreciable reduction of cell viability up to 5 µM antibody concentration (Fig. 1e). Similar results were obtained with hGBM-82 patient-derived cell cultures (Fig. S3). These findings demonstrate the antibody’s ability to target and inhibit GBM cell invasiveness in vitro.

### abEC1.1 targets Cx HCs in patient-derived GBM cell cultures

**Fig. 2:**
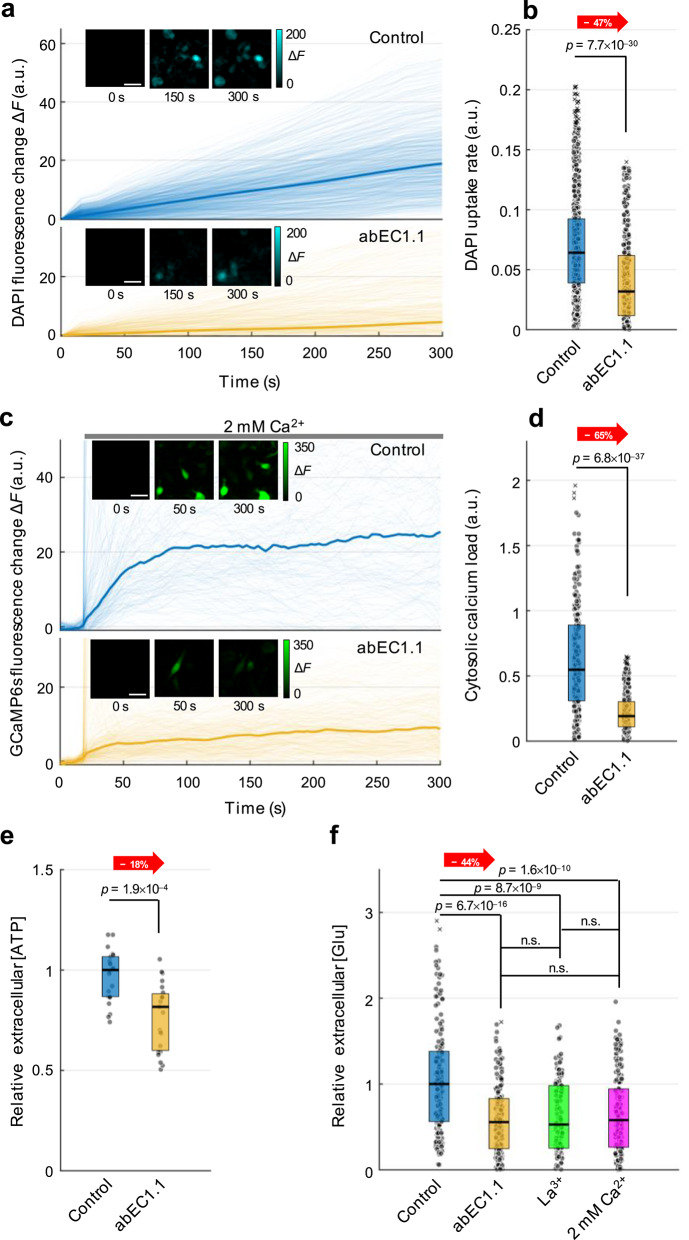
HC functionality assays in patient-derived hGBM-13 cell cultures. **a** Median DAPI fluorescence intensity (thick lines), overlaid with individual cell responses (light lines). Cells were maintained in ZCM (Control, upper panel; *n* = 820 cells, pooled data from 5 independent experiments) or ZCM containing 1 μM abEC1.1 (abEC1.1, lower panel; *n* = 350 cells, pooled data from 5 independent experiments). Insets show representative sequences of fluorescence images acquired at the indicated time points (corresponding to the x-axes of the relative plots); scale bar: 50 µm. **b** Dot-box plots showing DAPI uptake rates obtained from data in (**a**) by fitting DAPI fluorescence change Δ*F* vs. time with straight lines through the origin for each condition; *P*-values (*p*) determined by KW test. **c** Median GCaMP6s Δ*F* traces (thick lines) in response to a step increase in [Ca^2+^]_ex_ from 0 to 2 mM, overlaid with individual cell responses (light lines). Cells were maintained in ZCM (Control, upper panel; *n* = 170 cells, pooled data from 3 independent experiments) or ZCM containing 1 μM abEC1.1 (abEC1.1, lower panel; *n* = 350 cells, pooled data from 4 independent experiments). Insets show representative sequences of fluorescence images acquired at the indicated time points (corresponding to the x-axes of the relative plots); scale bar: 50 µm. **d** Dot-box plots showing cytosolic calcium load measured as the area under Δ*F* traces in (**c**) from *t* = 20 s to *t* = 300 s for each condition; *P*-values (*p*) determined by KW test. **e** Quantification of ATP release by luciferin-luciferase assay in control or 1 µm abEC1.1-treated hGBM-13 cell cultures (*n* = 23; pooled data from 3 independent experiments). **f** Quantification of glutamate (Glu) release by iGluSnFR fluorescence assay in: ZCM (Control, *n* = 170 cells, pooled data from 3 independent experiments); ZCM plus 1 µm abEC1.1 (*n* = 220 cells, pooled data from 3 independent experiments); ZCM plus 200 µm La^3+^ (*n* = 120 cells, pooled data from 3 independent experiments); ZCM plus 2 mM Ca^2+^ (*n* = 190 cells, pooled data from 3 independent experiments)

Regulation of HC gating by the extracellular free Ca^2+^ concentration ([Ca^2+^]_ex_) is at the core of widely used functional assays based on the cellular uptake of tracer molecules or Ca^2+^ through Cx HCs [96]. We therefore performed a series of experiments whereby patient-derived cells were exposed to an extracellular medium devoid of Ca^2+^ ions (ZCM) to increase the open probability of Cx HCs [35, 36] (Fig. 2 and Fig. S4). Compared to untreated controls, the median uptake rate of DAPI was significantly reduced (by 47%) in hGBM-13 cultures pre-incubated in ZCM supplemented with abEC1.1 (1 µM, 30 min; Fig. 2a, b).

To quantify Ca^2+^ uptake, patient-derived cells were virally transduced with a lentivirus expressing the GCaMP6s selective Ca^2+^ indicator [76, 78, 84]. Antibody treatment (1 µM, 30 min), significantly reduced (by 65%) the median cytosolic Ca^2+^ load (CCL) triggered by a step increase of the [Ca^2+^]_ex_ from 0 to 2 mM (Fig. 2c, d). Similar DAPI and Ca^2+^ uptake results were obtained with hGBM-82 cell cultures (Fig. S4a-d).

Given the importance of extracellular ATP and glutamate for GBM proliferation and invasion, we conducted additional assays to quantify the release of these critical molecules. With reference to median values, ATP release, probed by a luciferin-luciferase bioluminescence assay [34, 75, 78], was significantly reduced (by 18%) in hGBM-13 cultures (Fig. 2e) and (by 57%) in hGBM-82 cultures (Fig. S4e) treated with abEC1.1 (1 µM, 2 h). Glutamate release, probed by iGluSnFr fluorescence, was similarly reduced (by 44%) in hGBM-13 cultures (Fig. 2f) and (by 81%) in hGBM-82 cultures (Fig. S4f). These findings collectively suggest that the antibody limits glioma invasion in vitro by targeting functional Cx HCs expressed in the plasma membrane of patient-derived cells.

### abEC1.1 gene delivery is effective in vivo in the GL261 mouse model

**Fig. 3:**
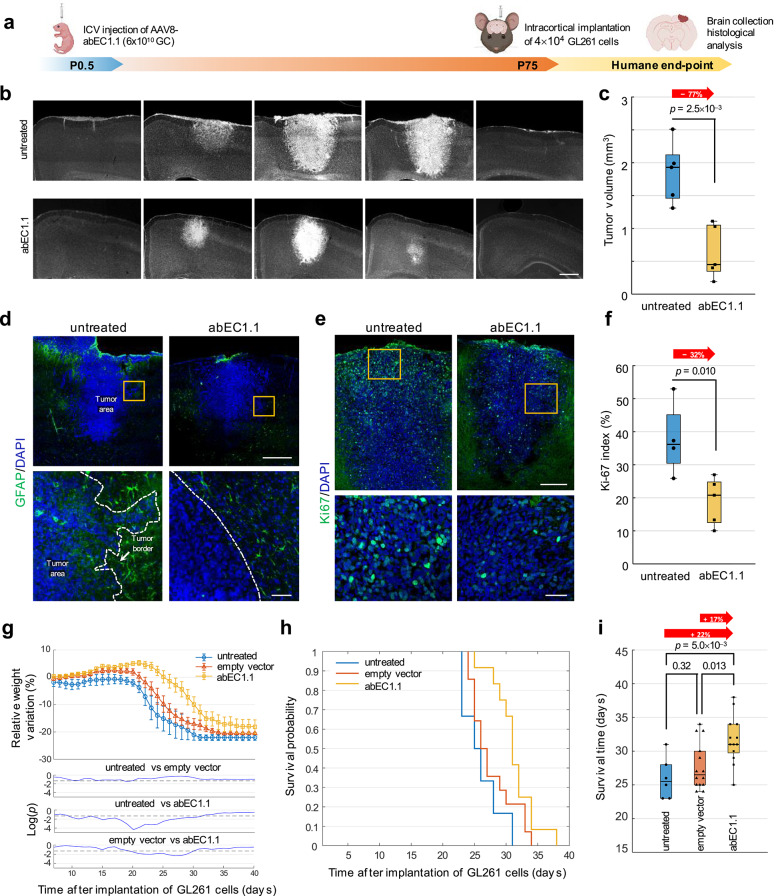
Antibody gene delivery reduces tumor growth and invasiveness and prolongs survival in the GL261 mouse model of GBM. **a** Schematic diagram illustrating the experimental pipeline. **b** DAPI staining (light grey) of coronal brain slices from mice treated with AAV8-abEC1.1 and corresponding untreated controls. Images refer to sections taken at approximately 250 µm intervals around the GL261 cell injection site from brains explanted 15 days after implantation of 4×10^4^ GL261 cells. Scale bar: 500 µm. Images are representative of *n* = 5 mice for each experimental group. **c** Dot-box plots of tumor volume from data related to (**b**). **d** Expression of Gfap (green) in coronal sections of tumor areas from untreated controls or AAV8-abEC1.1 injected mice; scale bars: 500 µm (upper panels); 50 µm (lower panels). **e** Expression of Ki-67 (green) in coronal sections in tumor areas from mice treated with AAV8-abEC1.1 and corresponding untreated controls; scale bar: 250 µm (upper panels); 50 µm (lower panels). Images in (**d**) and (**e**) are representative of *n* = 5 mice treated with AAV8-abEC1.1 and *n* = 4 untreated controls. **f** Dot-box plots quantification of Ki-67-positive nuclei from data related to (**e**). **g** Time course of the relative weight variation following intracortical implantation of 6×10^4^ GL261 cells in mice treated with ICV injection of AAV8-abEC1.1 at P0.5 (*n* = 13 mice, orange) or AAV8-empty (*n* = 14 mice, red) compared to untreated controls (*n* = 6 mice, blue). The three bottom graphs show point-by-point logarithmic plots of *P*-values (*p*) computed between each pair of experimental groups; the dashed lines indicate *p* = 0.05. **h** Kaplan-Meyer curves derived from data in (**g**) (orange, AAV8-abEC1.1; red, AAV8-empty; blue, untreated). **i** Dot-box plots of survival time after implantation of GL261 cells derived from data in (**h**)*. P*-values for all data in this figure were derived from the Wilcoxon rank sum test

Next, we evaluated the efficacy of antibody treatments in the GL261 orthotopic syngeneic mouse model of GBM [97, 98]. In the first set of these in vivo experiments (Fig. 3, Fig. S5 and Fig. S6), C57BL/6 J mice of the treatment group were administered 6 × 10^10^ gc of AAV8-abEC1.1 [77] via ICV injection at P0.5 [99] (Fig. S5a). This method allows endogenous production and secretion of the antibody by virally transduced neurons and astrocytes (Fig. S5 and Fig. S6), offering a long-lasting and cost-effective therapeutic strategy [100]. C57BL/6 J mice of control groups received either 6 × 10^10^ gc of AAV8-empty capsids, or no treatment. At P30, confocal immunofluorescence analysis of coronal brain sections labelled with an anti-mFc antibody confirmed expression of the secreted antibody in several brain areas of the treatment group, including cortex, hippocampus and cerebellum (Fig. S5b). At P75, mice of all groups were implanted intracortically with 4 × 10^4^ GL261 cells (Fig. S6a), weighed daily, and sacrificed either at P90, for brain section analyses, or at the humane endpoint (Fig. 3a).

At P90, confocal immunofluorescence analysis with anti-Cx selective antibodies localized Cx26 and Cx32 primarily in the peritumoral zone, Cx46 within the tumor mass, Cx30 and Cx43 in both regions (Fig. S6b). Additional experiments demonstrated colocalization of abEC1.1, labelled with an ALFA-tag, with Cx26 (Fig. S6c, d). Notably, the commercial antibodies used recognized intracellular Cx epitopes in fixed and permeabilized cells, while abEC1.1 targeted extracellular epitopes in intact Cx-expressing cells [73–78].

In mice treated with AAV8-abEC1.1, quantitative analysis of serial coronal brain sections stained with DAPI revealed significantly reduced tumor volumes (by 77%) compared to untreated controls (Fig. 3b, c). Treated animals also exhibited a more rounded peritumoral zone profile (consistent with the results of Fig. 1c), reduced density of tumor-associated Gfap-positive reactive astrocytes [101], and absence of necrotic areas within the tumor mass (Fig. 3d). These changes were accompanied by a significantly reduced (by 32%) Ki-67 proliferation index (Fig. 3e, f) [102].

Mice treated with AAV8-abEC1.1 experienced significantly slower body weight loss compared to controls (Fig. 3g). Kaplan–Meier survival analysis [103] (Fig. 3h) showed a significant increase (by 22%) of the median survival time (31 days) compared to untreated controls (25.5 days) (Fig. 3i). In contrast, mice treated with AAV8-empty capsids exhibited survival times (26.5 days) indistinguishable from those of untreated controls. These findings suggest that antibody gene delivery with adeno-associated viral vectors effectively impairs GBM progression in vivo.

### CED of abEC1.1 is effective in vivo in the GL261 mouse model

**Fig. 4:**
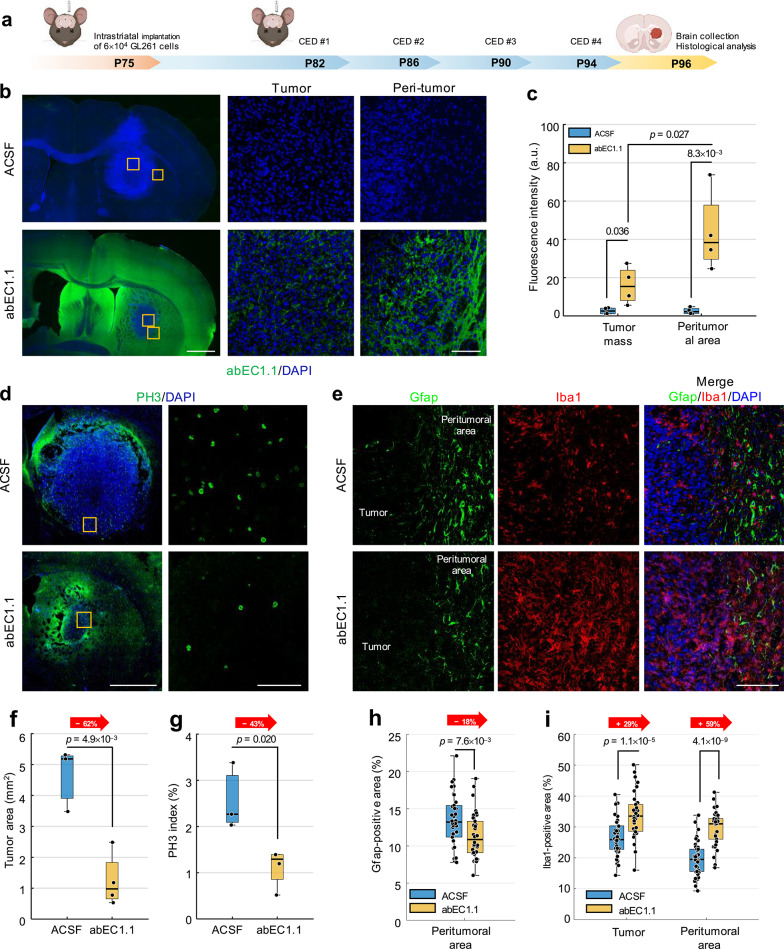
CED of the purified antibody reduces tumor growth and invasiveness in the GL261 mouse model of GBM. **a** Schematic diagram illustrating the experimental pipeline. **b** abEC1.1 distribution (green) in brain coronal sections of C57BL/6 J mice implanted with GL261 cells and treated with intratumoral CED of purified abEC1.1 antibody, labelled with an ALFA-tag, or saline; scale bars: 1 mm (left panel); 100 µm (right panels). Images are representative of n = 4 mice for each experimental group. **c** Dot-box plot quantification of anti-Atag antibody fluorescence intensity from data related to (**b**). **d** Expression of PH3 (green); scale bars: 1 mm (left panels); 100 µm (right panels). Images are representative of n = 4 mice treated with CED of purified abEC1.1 and n = 3 untreated controls. **e** Expression of Gfap (green) and Iba1 (red); scale bar: 100 µm. Images are representative of n = 4 mice treated with CED of purified abEC1.1 and n = 4 untreated controls. **f**, **g** Dot-box plot quantification of tumor area extent (**f**) and PH3 positive cells (**g**) from data related to (**d**). **h**, **i** Dot-box plot quantification of Gfap (**h**) and Iba1 (**i**) signals from data related to (**e**). P-values for all data in this figure were derived from the Wilcoxon rank sum test

Given the interest in CED for GBM treatment [104], we tested also this approach in a second set of in vivo experiments using abEC1.1 (Fig. 4 and Fig. S7). The antibody, labelled with an ALFA-tag, was dissolved at concentration of 5 mg/mL in ACSF and delivered into the striatum of healthy C57BL/6 J mice via CED at P75. Biodistribution analysis carried out in serial coronal brain sections revealed intense anti-ALFA-tag immunoreactivity in the injection region 2 h post-CED, and a widespread brain distribution by day 7, with an 88% intensity reduction in the striatum (Fig. S7). Based on these biodistribution results, another group of mice received 6 × 10^4^ GL261 cells in the striatum at P75, followed by CED of abEC1.1 at P82, P86, P90, and P94, with sacrifice at P96 (48 h after the last treatment) (Fig. 4a). Mice of the corresponding control group received vehicle alone (ACSF). At P96, analysis of serial coronal brain sections showed anti-ALFA-tag immunostaining not only in the peritumoral area but also in the tumor mass of treatment group animals (Fig. 4b, c). We quantified expression levels of the phospho-histone H3 (PH3) proliferation index [105] (Fig. 4d), Gfap [101] and Iba1, a marker of activated microglia/macrophages [106] (Fig. 4e). Compared to control group, treated mice showed a 62% reduction of tumor area at the injection site (Fig. 4f), a 43% reduction of the PH3 index within the tumor mass (Fig. 4g), and an 18% reduction of Gfap immunoreactivity in the peritumoral area (Fig. 4h). Conversely, Iba1 immunoreactivity increased by 59% in the peritumoral area and 29% in the tumor mass (Fig. 4i), suggesting a potential immune-mediated anti-tumor response [107]. These findings provide further insight into the mechanism that subtend the antibody’s effects on GBM in vivo.

### abEC1.1 reduces tumor-induced neuronal hyperexcitability in co-cultures of astrocytes, neurons and GL261 cells

**Fig. 5:**
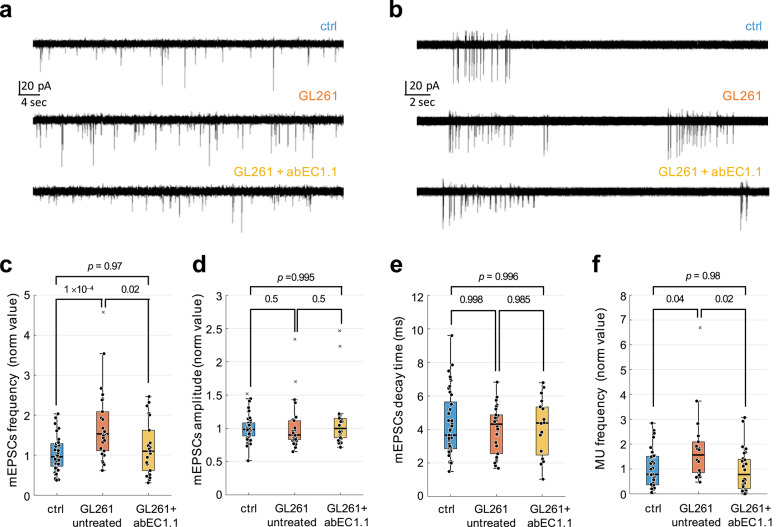
The antibody exerts anti-seizure control in co-cultures of astrocytes, neurons and GL261 cells. **a** Representative electrophysiological traces of mEPSCs recorded from neuron-astrocytes co-cultures (ctrl) and neuron-astrocytes-GBM (GL261) co-cultures treated or not (ctrl) with abEC1.1 (1 µM; *n* = 22) for 45 min. **b** Representative electrophysiological traces of MU activity recorded from neuron-astrocytes (ctrl) and neuron-astrocytes-GBM (GL261) co-cultures treated or not (ctrl) with abEC1.1 (1 µM) for 45 min. **c**–**e**. Dot-box plots of mEPSCs frequency (**c**), mEPSCs amplitude (**d**) and decay time (**e**) in the conditions shown. Number of neurons recorded in each condition: ctrl, *n* = 40; GL621, *n* = 26; Gl621 + abEC1.1, *n* = 22. **f** Dot-box plots of MU frequency in the experimental groups. Number of neurons recorded in each condition: ctrl, *n* = 38; GL621, *n* = 18; Gl621 + abEC1.1, *n* = 28. Each dot represents the mean value of the corresponding parameter estimated from a single neuron

To assess the impact of abEC1.1 on glioma-associated neuronal hyperexcitability, we established a 2D co-culture system comprising astrocytes and neurons isolated from mouse hippocampi, seeded with GL261 cells. Patch-clamp recordings [108] were performed from neurons 3 h after seeding (Fig. 5), as longer incubation with GL261 cells led to evident signs of neuronal degeneration, precluding later time-point recordings (Fig. S8). The frequency of miniature excitatory postsynaptic currents (mEPSCs, Fig. 5a) [109] and multi-unit (MU) spiking activity (Fig. 5b) [110] increased significantly in the presence of GL261 cells, reflecting aberrant synaptic activity. Treatment with abEC1.1 (1 µM) reduced both MU frequency and mEPSC frequency without altering mEPSC amplitude or decay time (Fig. 5c-f).

**Fig. 6:**
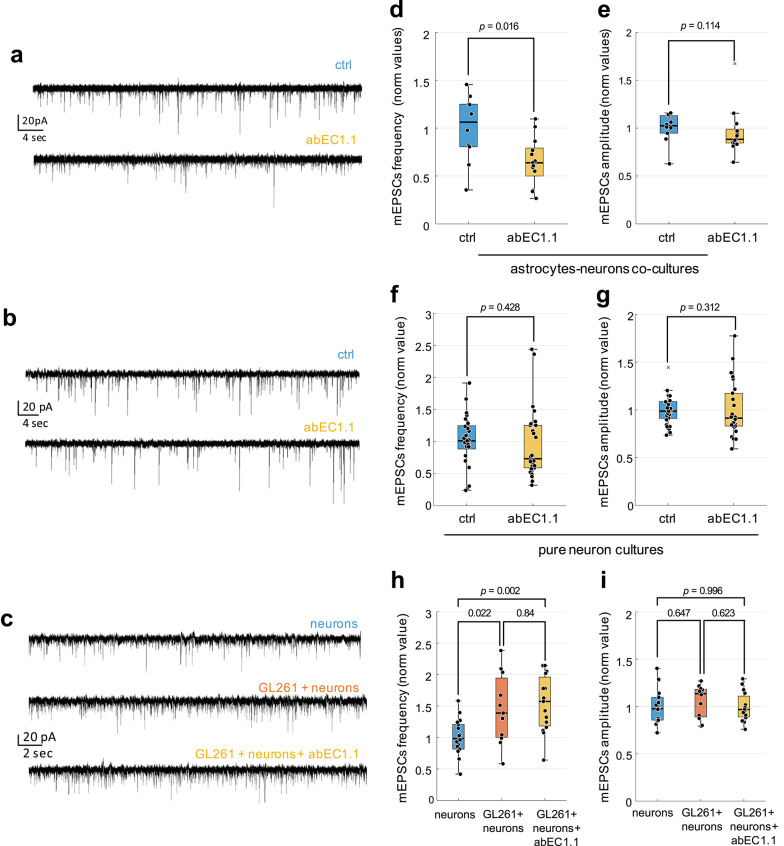
The effect of the antibody depends on the presence of astrocytes in the co-culture model. **a, b, c** Representative electrophysiological traces of mEPSCs recorded from astrocytes-neurons co-cultures (**a**), pure DIV14 hippocampal neurons (**b**), and GBM-neurons co-cultures (**c**) exposed to vehicle (ctrl) or abEC1.1 (1 µM) for 45 min. **d**, **e** Dot-box plots of mEPSCs frequency (**d**) and amplitude (**e**) in astrocytes-neurons co-cultures. Number of neurons recorded in each condition: ctrl, *n* = 10; abEC1.1, *n* = 13. **f**, **g** Dot-box plots of mEPSCs frequency (**f**) and amplitude (**g**) in pure neuronal cultures. Number of neurons recorded in each condition: ctrl, *n* = 29; abEC1.1, *n* = 26. Each dot represents the mean value of the corresponding parameter estimated from a single neuron. **h**, **i** Dot-box plots of mEPSCs frequency (**h**) and amplitude (**i**) in GBM-neurons co-cultures. Number of neurons recorded in each condition: neurons, *n* = 18; ctrl, *n* = 11; abEC1.1, *n* = 15

To directly show that the control of synaptic activity by abEC1.1 was mediated through astrocytic Cxs, we recorded mEPSC events in three different co-cultures conditions: i) astrocyte-neuron co-cultures (Fig. 6a), ii) pure neuronal cultures (Fig. 6b), and iii) GBM-neuron co-cultures (Fig. 6c). The frequency of mEPSCs (Fig. 6d), but not their amplitude (Fig. 6e), was significantly reduced by 1 µM abEC1.1 in astrocyte-neuron co-cultures, whereas no changes of these parameters were observed in either pure neuronal hippocampal cultures (Fig. 6f, g) or in GBM-neurons co-cultures (Fig. 6h, i). Altogether, these findings suggest that GL261 cells increase glutamatergic activity also through a direct effect on neurons and that abEC1.1 reduces glioma-induced excitatory synaptic activity by selectively blocking astrocytic Cxs, supporting its potential as an anti-seizure therapy in patients affected by glioma.

## Discussion

The results presented in this study highlight the functional state of Cx HCs within tumors in vivo and underscore the therapeutic potential of the abEC1.1 antibody in targeting Cx HCs in GBM. This approach offers a promising strategy to disrupt the molecular and cellular networks driving GBM progression, invasiveness, and therapy resistance. Below, we discuss the implications of these findings in the context of GBM pathophysiology and potential clinical applications.

### Targeting Cx HCs: A promising therapeutic avenue

Cx HCs are normally subject to strict regulation by various factors such as phosphorylation, redox balance, pH, extra- and intra-cellular Ca^2+^ levels. However, in glioma/GBM, these control mechanisms are often impaired due to the altered TME [111], and a multifaced interplay among Ca^2+^, glutamate and purinergic signaling involving glioma and immune cells, astrocytes, neurons, vasculature and extracellular matrix [22], potentially leading to enhanced HC activity [71]. In this study, we show a clear effect in patient-derived GBM cultures, where abEC1.1 significantly limited DAPI and Ca^2+^ uptake, two well-established assays of HC functionality. Our findings also confirm that abEC1.1 significantly reduced ATP and glutamate release. ATP supports tumor migration by activating purinergic receptors, while glutamate release promotes excitotoxicity, leading to neuronal damage and enhanced tumor expansion. Additionally, Ca^2+^ signaling regulates key downstream pathways such as MAPK and NF-κB, which drive proliferation, invasion, and resistance to apoptosis. By attenuating these signals, abEC1.1 has shown potential to directly impact the molecular mechanisms underpinning GBM aggressiveness, as further supported by results from our scratch and invasion assays. Mechanistically, the inhibitory effects of abEC1.1 depend on its binding to critical amino acids (N54, T55, L56, Q57, P58, P175 and N176) that are conserved in the extracellular domain of Cx26, Cx30 and Cx32, resulting in a sizeable reduction of permeant ions and molecules fluxes through the open HC pore [72–74].

Recent findings have demonstrated that Cx43 facilitates GBM invasion via extracellular vesicles (EVs), reinforcing the importance of this Cx in tumor progression. However, as shown also here, human GBM samples, as well as TME structures critically involved in GBM progression, such as peritumoral astrocytes, blood vessels and infiltrating microglia, express various other Cx isoforms. By inhibiting Cx26, Cx30, and Cx32 HCs, abEC1.1 expands its therapeutic scope beyond EV-mediated invasion, addressing multiple mechanisms simultaneously.

### In vivo efficacy of abEC1.1 gene delivery and CED

Our findings demonstrate that AAV8-mediated gene delivery of abEC1.1 in the GL261 mouse model provides an effective in vivo approach for targeting Cx HCs. Previously, we successfully employed gene delivery of this antibody to target Cx HCs in a model of hereditary skin disease [77]. Here, we show that tumor volume reduction, decreased proliferation, and extended survival highlight therapeutic potential of the antibody in GBM. Importantly, these effects were accompanied by reduced Gfap-positive reactive astrocytes [101]. These and other microenvironmental cells support tumor progression and resistance by providing survival-promoting metabolites like glutathione, which protect GBM cells from oxidative stress and therapeutic interventions [112]. We and others have shown that HCs targeted by abEC1.1, such as Cx26 and Cx30, are conduits for glutathione release [113, 114].

The observed reduction in tumor necrosis following abEC1.1 treatment is also significant, as necrotic regions often foster immunosuppressive and pro-tumorigenic microenvironments through factors like hypoxia, release of damage-associated molecular patterns (DAMPs), and recruitment of immunosuppressive cells such as regulatory T cells and myeloid-derived suppressor cells (MDSCs) [81, 115–117].

CED delivery of abEC1.1 further validated the feasibility of direct antibody administration to GBM [104], achieving significant reductions in tumor area, Gfap expression [101], and PH3 levels [105], complementing other proliferation markers such as Ki-67 [102]. Furthermore, increased Iba1 expression in treated animals suggests activation of microglia and macrophages [106]. Given emerging evidence that Cx signaling modulates the tumor immune microenvironment [118], further studies should explore the interplay between abEC1.1 treatment and immune activation.

### In vitro efficacy of abEC1.1 against tumor-induced neuronal hyperexcitability

Aberrant glutamate release has long been implicated in both glioma invasion [49–52] and glioma-associated neuronal hyperexcitability, which is driven by tumor-induced disruption of the excitatory-inhibitory (E/I) balance [53–57]. Growing evidence suggests astrocytes and astrocytic Cxs actively influence epileptic activity and seizure outcomes [58–62]. Given that astrocytes release glutamate through unpaired Cx HCs [46–48], we hypothesized that manipulating astrocytic Cx HCs may counteract the aberrant glutamate release and glioma-related hyperexcitability. Our results demonstrate that abEC1.1 reduced mEPSC frequency in astrocyte-neuron co-cultures but neither in pure neuronal cultures, nor in GBM-neurons co-cultures, confirming its selective inhibition of astrocytic Cx HCs. Further validation through MU activity recordings revealed abEC1.1 normalized hyperexcitability in neuron-astrocyte co-cultures overlaid with GL261 cells. Our findings therefore suggest that abEC1.1-mediated inhibition of neuronal hyperactivity may provide dual benefits: reducing both seizure burden and tumor growth.

### Therapeutic implications

The tight connection between neuronal hyperexcitability and glioma invasion has prompted the search for anticonvulsants with cytostatic properties [55, 56]. Valproic acid, for example, enhances glioma chemosensitivity via histone acetylation [119], while TMZ has demonstrated anti-epileptic effects [120]. The antiseizure drug perampanel has shown antitumor activity in vitro; however, no evidence has been found to support its role in slowing tumor progression in rat models. Additionally, clinical data on perampanel’s potential antitumor effects remain limited [121]. In this context, abEC1.1 is a promising candidate which can simultaneously inhibit tumor growth and suppress glioma-induced excitatory activity without altering synaptic function in healthy neurons. Future research should validate these findings in vivo by assessing whether abEC1.1 reduces seizure incidence and slows tumor progression in glioma-bearing mouse models.

### Limitations and future directions

While the findings are promising, several limitations must be addressed. First, the variability in Cx expression across GBM subtypes and patient-derived cultures necessitates a more comprehensive analysis of abEC1.1 efficacy across a broader spectrum of GBM models. Second, although abEC1.1 showed efficacy in the GL261 mouse model, its translational potential in human GBM requires further validation in orthotopic patient-derived xenograft models [122]. Third, all electrophysiological experiments were limited to a 3 h co-culture window, as neuronal degeneration and death were observed as early as 6 h after GL261 seeding. While this early time point allowed us to capture acute glioma-induced synaptic alterations, it does not fully recapitulate the chronic and dynamic interactions occurring in the TME in vivo. Future studies employing more stable systems may help extend the observation window and better reflect long-term neuron–glioma interactions.

Additionally, although our in vitro data suggest a safe profile of abEC1.1, its impact on normal brain physiology warrants investigation. Cxs are widely expressed in healthy tissues, where they play critical roles in maintaining cellular homeostasis. Strategies to optimize abEC1.1’s specificity for tumor-associated connexins, such as targeting extracellular epitopes unique to GBM, could enhance therapeutic efficacy while minimizing off-target effects.

Finally, the potential for combination therapies should be explored [123]. Disrupting Cx-mediated tumor networks may sensitize GBM to standard therapies, including radiotherapy and TMZ, as well as emerging treatments such as immune checkpoint inhibitors. Preclinical studies combining abEC1.1 with these modalities could provide valuable insights into synergistic effects and optimal therapeutic regimens. The safety of long-term Cx HC inhibition in non-tumor astrocytes must be rigorously evaluated to ensure minimal impact on physiological functions.

## Conclusions

Our findings support abEC1.1 as a complementary approach to Cx43-specific treatments, given its broader range of molecular targets and ability to interfere with multiple glioma invasion mechanisms. Future research should further explore its effects in combination therapies and validate its efficacy in additional preclinical GBM models.

## Supplementary Information

Supplementary Material 1

Supplementary Material 2

## Data Availability

No datasets were generated or analysed during the current study.

## Abbreviations

AAV: Adeno-Associated Virus
ACSF: Artificial Cerebrospinal Fluid
ATP: Adenosine Triphosphate
bFGF: Basic Fibroblast Growth Factor
Ca^2+^: Ionized Calcium
CED: Convection-Enhanced Delivery
Cx: Connexin
EGF: Epidermal Growth Factor
EGTA: Ethyleneglycol-bis(β-aminoethyl)-N,N,Nʹ,Nʹ-tetraacetic Acid
DAPI: 4′,6-Diamidino-2-phenylindole
DAMPs: Damage-associated molecular patterns
GBM: Glioblastoma
GSCs: Glioma stem cells
HC: Hemichannel
KW: Kruskal-Wallis
ICV: Intracerebroventricular
mEPSCs: Miniature excitatory postsynaptic currents
MGMT: O-6-methylguanine-DNA methyltransferase
MDSCs: Myeloid-derived suppressor cells
MU: Multi-Unit
P: Postnatal day
RO: Retro-orbital
TME: Tumor microenvironment
TMZ: Temozolomide
