# Supplementary material for: Connexin hemichannel blockade by abEC1.1 disrupts glioblastoma progression, suppresses invasiveness, and reduces hyperexcitability in preclinical models
Source: Cell Commun Signal. 2025 Sep 2;23:391. doi: 10.1186/s12964-025-02370-1 (PMC12403430; doi:10.1186/s12964-025-02370-1)
Supplement: Supplementary file 1 — Supplementary Material 1 [file 12964_2025_2370_MOESM1_ESM.pdf]

Figure S1

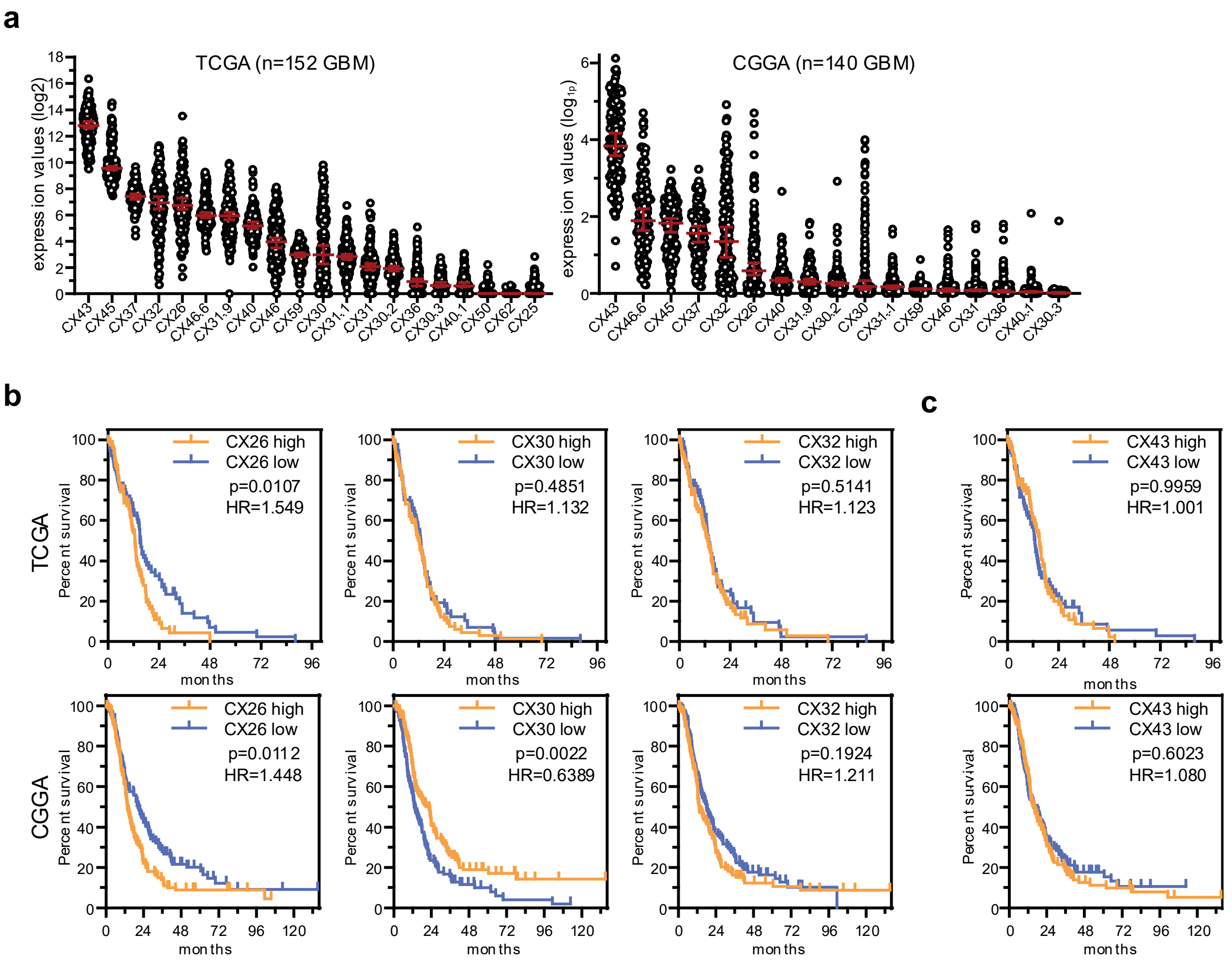
a
TCGA (n=152 GBM)
expression values (log2)
CX43 CX45 CX37 CX32 CX26 CX46.6 CX31.9 CX40 CX46 CX59 CX30 CX31.1 CX31 CX30.2 CX36 CX30.3 CX40.1 CX50 CX62 CX25
CGGA (n=140 GBM)
expression values (log1p)
CX43 CX46.6 CX45 CX37 CX32 CX26 CX40 CX31.9 CX30.2 CX30 CX31.1 CX59 CX46 CX31 CX36 CX40.1 CX30.3
b
TCGA
CGGA
Percent survival
months
CX26 high
CX26 low
p=0.0107
HR=1.549
CX30 high
CX30 low
p=0.4851
HR=1.132
CX32 high
CX32 low
p=0.5141
HR=1.123
CX26 high
CX26 low
p=0.0112
HR=1.448
CX30 high
CX30 low
p=0.0022
HR=0.6389
CX32 high
CX32 low
p=0.1924
HR=1.211
c
CX43 high
CX43 low
p=0.9959
HR=1.001
CX43 high
CX43 low
p=0.6023
HR=1.080

Figure S2

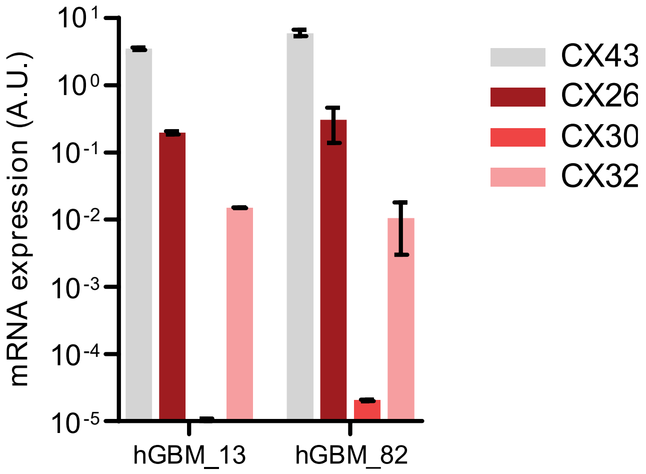
mRNA expression (A.U.)
10^1
10^0
10^-1
10^-2
10^-3
10^-4
10^-5
hGBM_13
hGBM_82
CX43
CX26
CX30
CX32

Figure S3

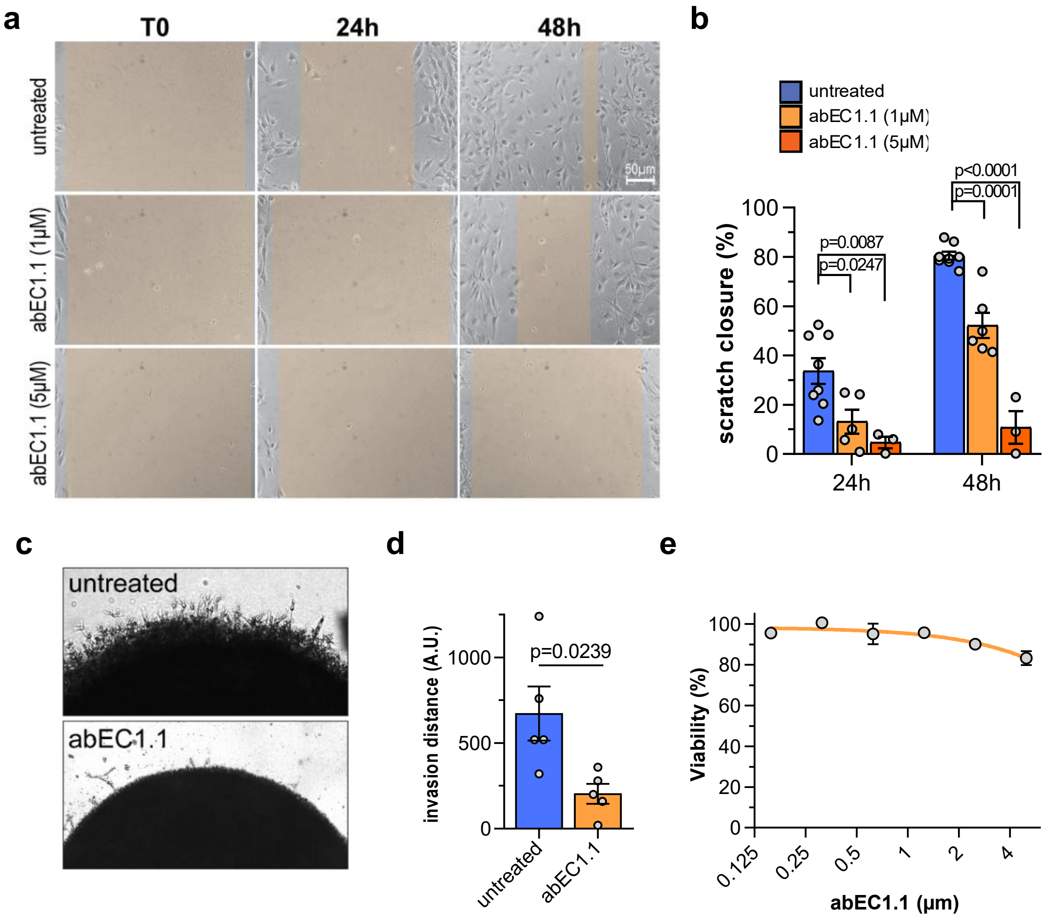
a
T0
24h
48h
untreated
abEC1.1 (1μM)
abEC1.1 (5μM)
50μm
b
untreated
abEC1.1 (1μM)
abEC1.1 (5μM)
p<0.0001
p=0.0001
p=0.0087
p=0.0247
scratch closure (%)
100
80
60
40
20
0
24h
48h
c
untreated
abEC1.1
d
invasion distance (A.U.)
p=0.0239
1000
500
0
untreated
abEC1.1
e
Viability (%)
100
80
60
40
20
0
0.125
0.25
0.5
1
2
4
abEC1.1 (μm)

Figure S4

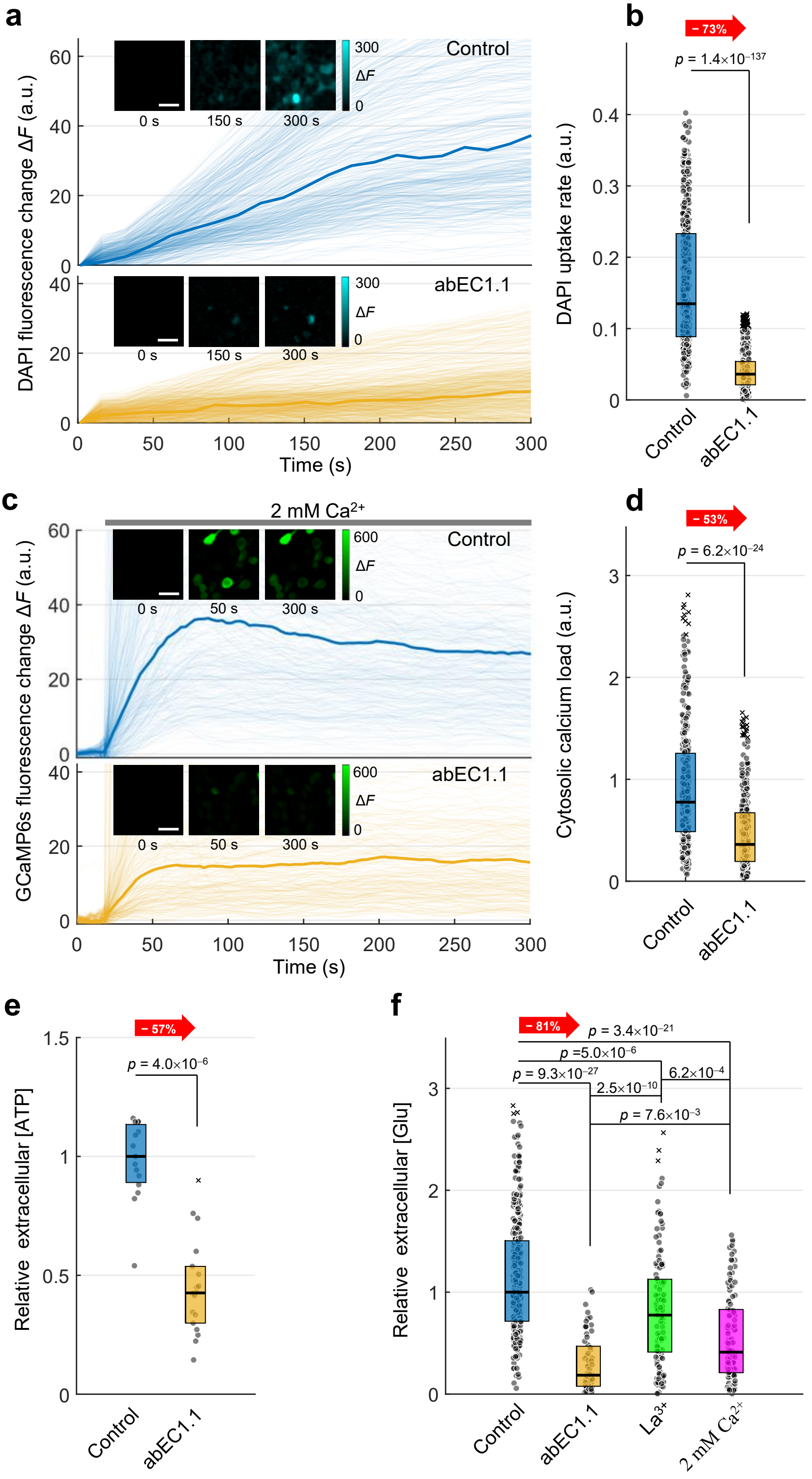

a
DAPI fluorescence change ΔF (a.u.)
Control
abEC1.1
0 s
150 s
300 s
300
ΔF
0
Time (s)
b
– 73%
p = 1.4×10−137
DAPI uptake rate (a.u.)
Control
abEC1.1
c
2 mM Ca2+
GCaMP6s fluorescence change ΔF (a.u.)
Control
abEC1.1
0 s
50 s
300 s
600
ΔF
0
Time (s)
d
– 53%
p = 6.2×10−24
Cytosolic calcium load (a.u.)
Control
abEC1.1
e
– 57%
p = 4.0×10−6
Relative extracellular [ATP]
Control
abEC1.1
f
– 81%
p = 3.4×10−21
p =5.0×10−6
p = 9.3×10−27
6.2×10−4
2.5×10−10
p = 7.6×10−3
Relative extracellular [Glu]
Control
abEC1.1
La3+
2 mM Ca2+

Figure S5

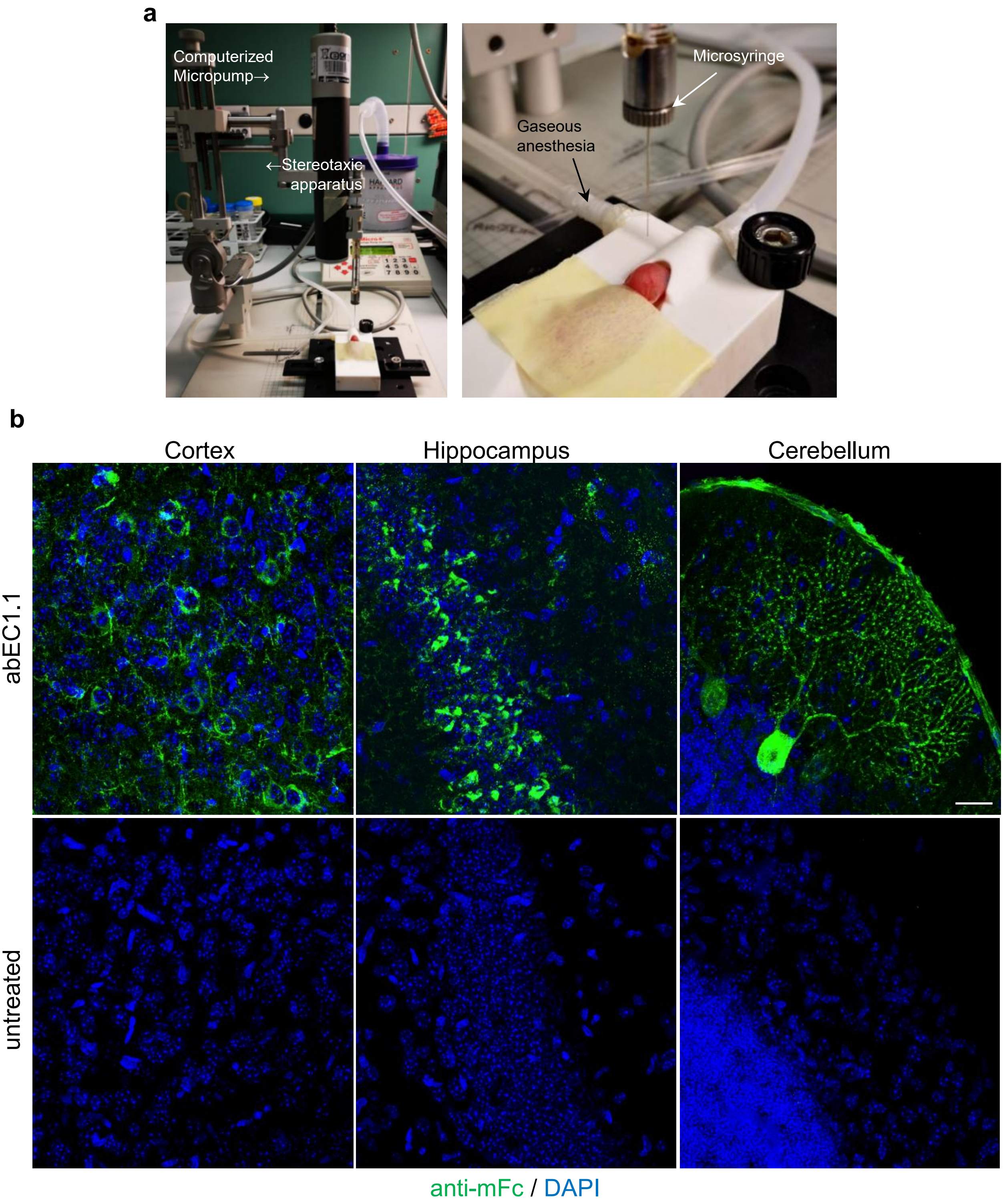
a
Computerized
Micropump→
←Stereotaxic
apparatus
Microsyringe
Gaseous
anesthesia
b
Cortex
Hippocampus
Cerebellum
abEC1.1
untreated
anti-mFc / DAPI

Figure S6

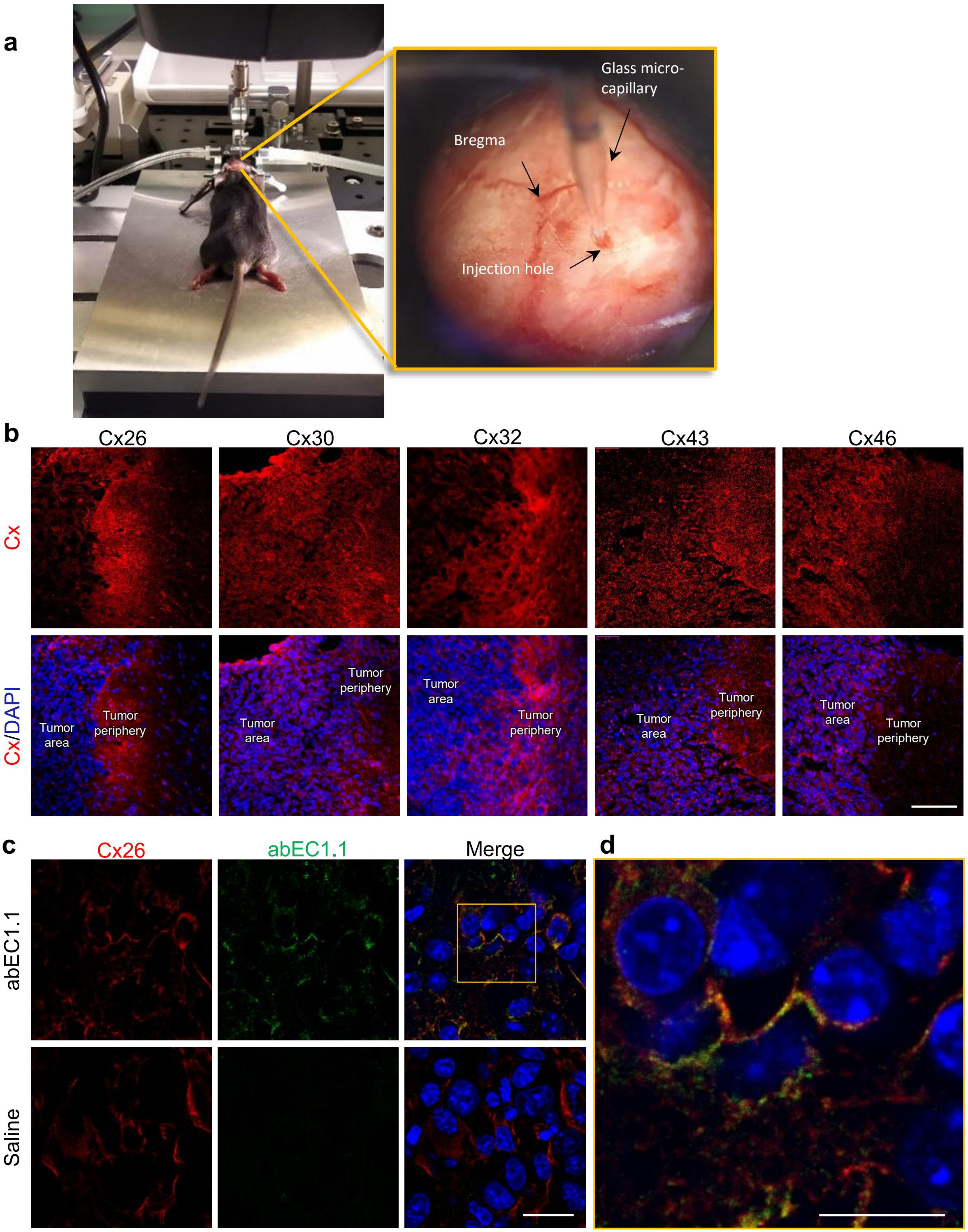

a
Glass micro-
capillary
Bregma
Injection hole
b
Cx26
Cx30
Cx32
Cx43
Cx46
Cx
Cx/DAPI
Tumor
area
Tumor
periphery
c
Cx26
abEC1.1
Merge
abEC1.1
Saline
d

Figure S7

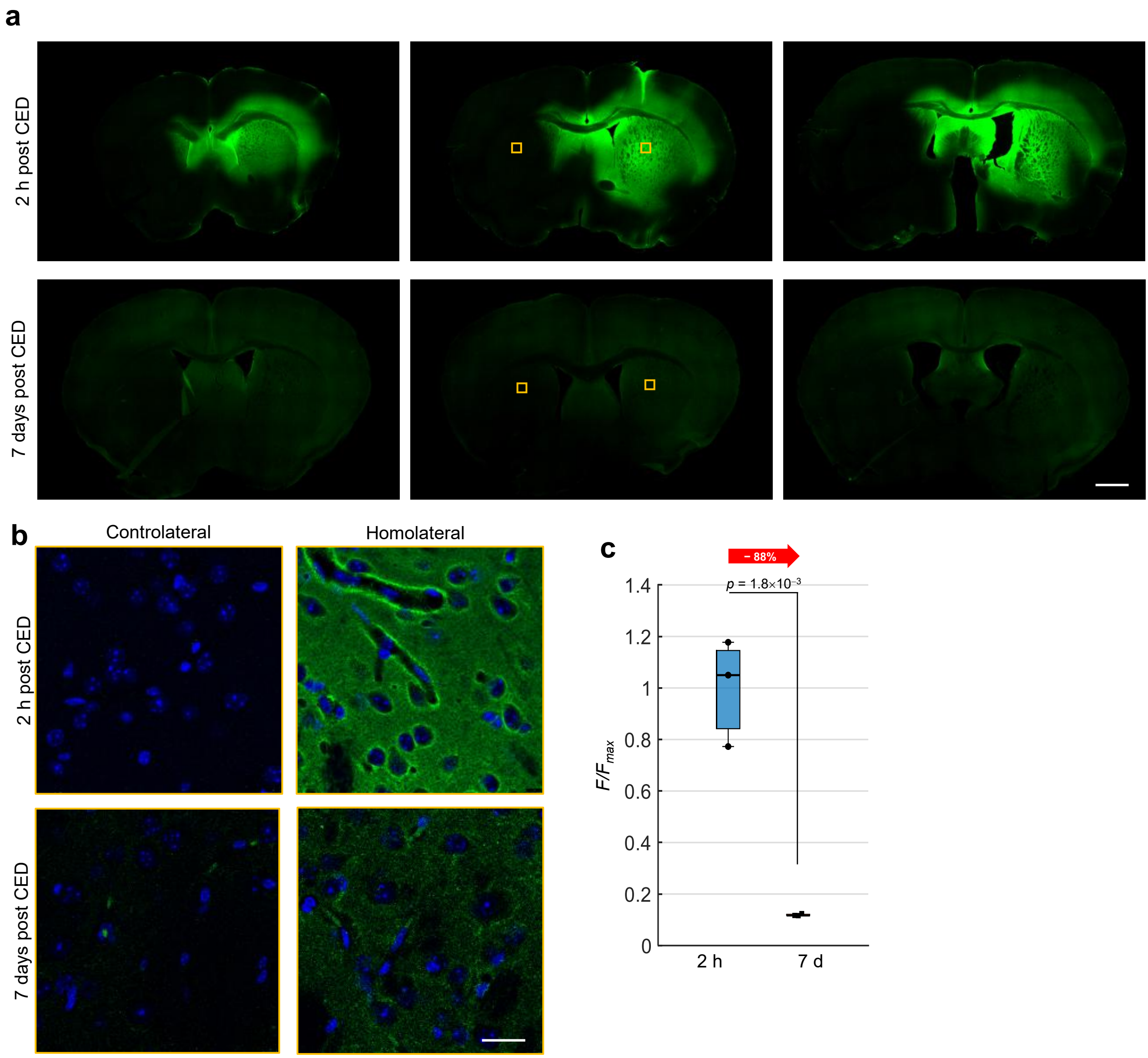

a
2 h post CED
7 days post CED
b
Controlateral
Homolateral
2 h post CED
7 days post CED
c
– 88%
p = 1.8×10⁻³
1.4
1.2
1
0.8
0.6
0.4
0.2
0
F/Fmax
2 h
7 d

Figure S8

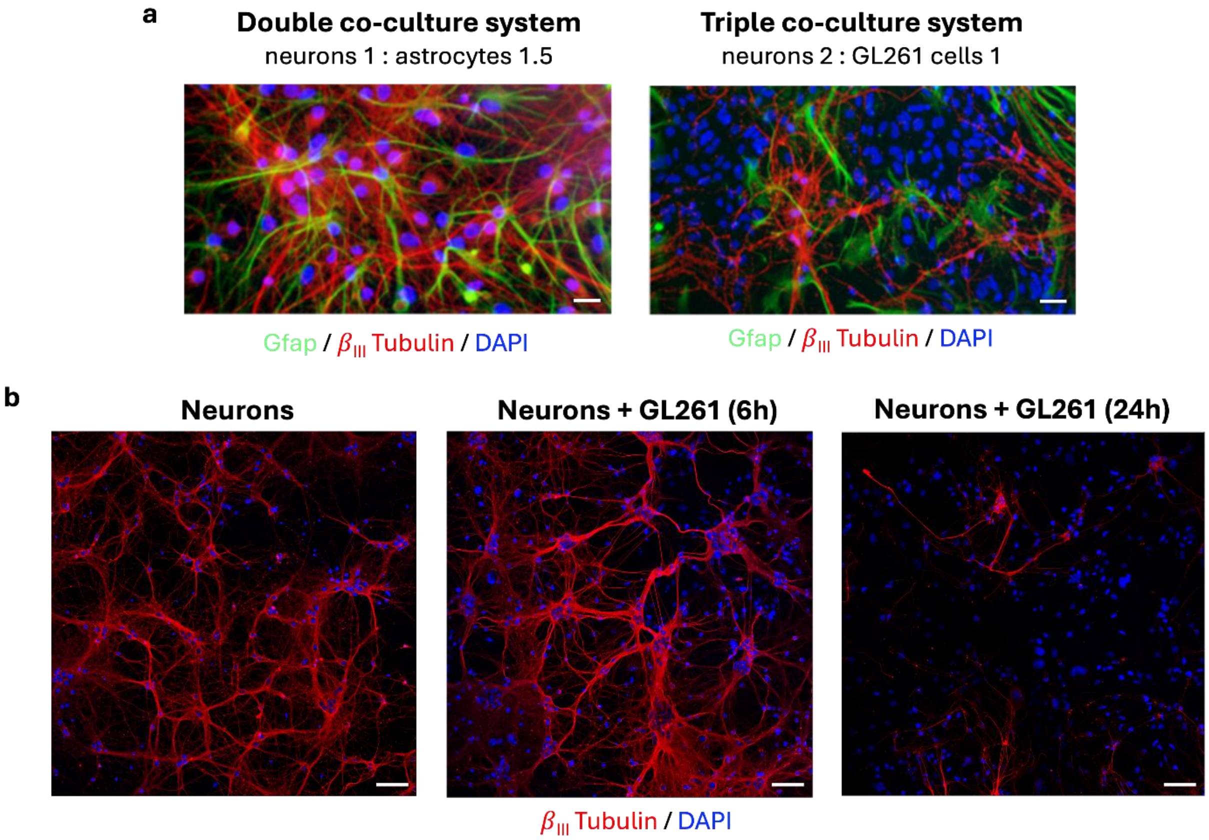

a
Double co-culture system
neurons 1 : astrocytes 1.5
Gfap / β_{III} Tubulin / DAPI
Triple co-culture system
neurons 2 : GL261 cells 1
Gfap / β_{III} Tubulin / DAPI
b
Neurons
Neurons + GL261 (6h)
Neurons + GL261 (24h)
β_{III} Tubulin / DAPI
