# Supplementary material for: Connexin hemichannel blockade by abEC1.1 disrupts glioblastoma progression, suppresses invasiveness, and reduces hyperexcitability in preclinical models
Source: Cell Commun Signal. 2025 Sep 2;23:391. doi: 10.1186/s12964-025-02370-1 (PMC12403430; doi:10.1186/s12964-025-02370-1)
Supplement: Supplementary file 2 — Supplementary Material 2 [file 12964_2025_2370_MOESM2_ESM.pdf]

Supplementary figure legends

Fig. S1 Dataset-derived results.

a Interrogation of the TCGA and CGGA RNAseq datasets for the expression of available *CX* genes in GBM tumors (grade IV). **b**, **c** Impact of *CX26*, *CX30*, *CX32* (B), and *CX43* (C) expression on the percent survival of GBM patients vs. time from the TCGA (upper panel) and CGGA (lower panel) datasets. P-values (*p*) calculated by Log-rank (Mantel-Cox) test. HR: Hazard Ratio. Both expression and survival data were retrieved from GlioVis, https://gliovis.bioinfo.cnio.es/).

Fig. S2 Expression levels of *CX26*, *CX30*, *CX32* and *CX43* mRNA in patient-derived cell cultures.

mRNA expression level ($2^{-\Delta Ct}$ transformed) of CX26, CX30, CX32 and CX43 in hGBM-13 and hGBM-82 primary cultures.

Fig. S3 Scratch, invasion, and antibody toxicity assays in hGBM-82 cell cultures.

a Representative images displaying the effect exerted by increasing doses of abEC1.1 on the ability of hGBM-82 primary cells to close the scratch over time. Scale bar: 50 µm. **b** Bar-dot plots of percent scratch closure data from (**a**); pooled data from n=3 independent experiments. P-values (p) determined by One-way ANOVA with Dunnett' multiple comparisons test. **c** Representative images displaying the ability of control or abEC1.1 (1 µM)-treated hGBM-82 organoids to invade Matrigel in 48 h. **d** Bar-dot plots of cell invasion quantification, measured by the mean distance between invasive cells and organoid edges; pooled data from n=5 independent experiments. **e** Dose response curves showing the effect of the administration of scalar doses of abEC1.1 on hGBM-82 cell viability, through a resazurin-based assay.

Fig. S4 HC functionality assays in hGBM-82 cell cultures.

a Median DAPI fluorescence intensity (thick lines), overlaid with individual cell responses (light lines). Cells were maintained in ZCM (Control, upper panel; n=460 cells, pooled data from 3 independent experiments) or ZCM containing 1 µM abEC1.1 (abEC1.1, lower panel; n=680 cells, pooled data from 4 independent experiments). Insets show representative sequences of fluorescence images acquired at the indicated time points (corresponding to the x-axes of the relative plots); scale bar: 50 µm. **b** Dot-box plots showing DAPI uptake rates obtained from data in (**a**) by fitting DAPI fluorescence variation in time with straight lines through the origin for each condition; P-values (*p*) determined by KW test. **c** Median GCaMP6s ΔF traces (thick lines) in response to a step increase in

$[Ca^{2+}]_{ex}$ from 0 mM to 2 mM, overlaid with individual cell responses (light lines). Cells were maintained in ZCM (Control, upper panel; n=290 cells, pooled data from 3 independent experiments) or ZCM containing 1 μM abEC1.1 (abEC1.1, lower panel; n=310 cells, pooled data from 3 independent experiments). Insets show representative sequences of fluorescence images acquired at the indicated time points (corresponding to the x-axes of the relative plots); scale bar: 50 μm. **d** Dot-box plots showing cytosolic calcium load measured as the area under ΔF traces in (**C**) from t=20 s to t=300 s for each condition; P-values (p) determined by KW test. **e** Quantification of ATP release by luciferin-luciferase assay in control or 1 μm abEC1.1-treated hGBM-82 cell cultures (n=18; pooled data from 3 independent experiments). **f** Quantification of glutamate (Glu) release by iGlusnFr fluorescence in: ZCM (Control, n=300 cells, pooled data from 4 independent experiments); ZCM plus 1 μm abEC1.1 (n=70 cells, pooled data from 3 independent experiments); ZCM plus 200 μm La^{3+} (n=125 cells, pooled data from 3 independent experiments); ZCM plus 2 mM Ca^{2+} (n=90 cells, pooled data from 3 independent experiments).

Fig. S5 Biodistribution of abEC1.1 following ICV administration of AAV8-abEC1.1 at P0.5.
Expression of abEC1.1 probed by an anti-mFc antibody (green) in various brain areas in sagittal sections of C57BL/6J mouse brains at P33. Nuclei were counterstained with DAPI (blue); scale bar: 10μm.

Fig. S6 Intracortical implantation of GL261 cells in C57BL/6J mice.
a Procedure. **b** Immunoreactivity to commercial antibodies selective for Cx26, Cx30, Cx32, Cx43 and Cx46 (red); scale bar: 100 μm. **c** Colocalization of Cx26 (red) and abEC1.1 (green) in mice retro-orbitally injected with AAV-PHP.eB-abEC1.1(scFv-mFc) or saline; scale bar: 20 μm. **d** Magnification of the boxed area in B; scalebar: 10 μm.

Fig. S7 Biodistribution of abEC1.1 after CED of the purified antibody.
a abEC1.1-mIgG1-Atag immunoreactivity (green) 2 h and 7 days after intrastriatal CED of the purified antibody; scale bar: 1 mm. **b** Magnified view of the boxed areas in **a**; scale bar: 25 μm. **c** Quantification of abEC1.1-mIgG1-Atag immunoreactivity normalized to the mean value measured at 2 h post CED (F_{max}). P-values (p) derived from the KW test.

Fig. S8 Characterization of neuronal–astrocytic and GBM–neuronal–astrocytic co-cultures.
a Representative confocal images of hippocampal neuron-astrocyte co-cultures in the absence (left; neuron:astrocyte ratio 1:1.5) or presence (right; neuron:GL261 ratio 2:1) of GL261 cells, 3 h after

GL261 seeding. Neurons are stained for β_{III}-tubulin (red), astrocytes for GFAP (green), and nuclei for DAPI (blue). Non-neuronal/non-astrocytic nuclei in the indicate GL261 cells. Neuronal degeneration is already detectable at this early time point in the presence of GL261 cells. Scale bars: 31 μm. **b** Representative images of β_{III}-tubulin$^+$ neurons (red) and DAPI$^+$ nuclei (blue) in neuronal cultures at different time points, in absence (left) or presence (center and right) of GL261 cells. Neuronal degeneration is already evident after 6 h of co-culture with GL261 cells and becomes dramatic at 24 h. Scale bars: 100 μm.
